# Natural Variation in *ZmASB1* Links Tryptophan Metabolism to Root Growth and Drought Tolerance in Maize

**DOI:** 10.1002/advs.202523671

**Published:** 2026-11-10

**Authors:** Xiaoyi Yang, Houmiao Wang, Xiaomin Zhang, Yunyun Wang, Yimei Tian, Baoqing Wang, Tianze Zhu, Shuai Fang, Wei Li, Wenye Rui, Yining Zhang, Dan Sun, Haoxin Gao, Yuxuan Li, Zhongtao Jia, Zefeng Yang, Chenwu Xu, Pengcheng Li

**Affiliations:** ^1^ Jiangsu Key Laboratory of Crop Genomics and Molecular Breeding/Key Laboratory of Plant Functional Genomics of the Ministry of Education Agriculture College of Yangzhou University Yangzhou China; ^2^ Jiangsu Co‐Innovation Center for Modern Production Technology of Grain Crops/Jiangsu Key Laboratory of Crop Genetics and Physiology Yangzhou University Yangzhou China; ^3^ State Key Laboratory of Crop Stress Adaptation and Improvement School of Life Sciences Henan University Kaifeng China; ^4^ National Observation and Research Station of Agriculture Green Development Quzhou China; ^5^ State Key Laboratory of Nutrient Use and Management College of Resources and Environmental Sciences China Agricultural University Beijing China

**Keywords:** anthranilate synthase, drought tolerance, maize, root architecture, tryptophan metabolism

## Abstract

Root amino acid metabolism is important for maize adaptation to water deficit, but the genetic mechanisms underlying this relationship remain poorly understood. We profiled 18 free amino acids in the roots of 192 diverse maize inbred lines and integrated genome‐wide association analyses with root transcriptome data. This approach identified 118 metabolite QTLs and prioritized *ZmASB1*, which encodes the β‐subunit of anthranilate synthase, as a candidate regulator of tryptophan (Trp) accumulation. A cis‐regulatory locus in the *ZmASB1* promoter was associated with variation in *ZmASB1* expression and root Trp abundance. Functional analyses indicated that *ZmASB1* promotes Trp accumulation and root growth through Trp‐dependent auxin biosynthesis. *ZmASB1*‐overexpressing lines showed enhanced root development and drought tolerance, whereas the mutant exhibited the opposite phenotypes. Exogenous Trp or auxin partially rescued the root‐growth and drought‐sensitive phenotypes of the mutant. We identified a 5‐bp insertion/deletion (InDel‐178) in the *ZmASB1* promoter that affects promoter activity by altering the binding of the transcription factor ZmDOF17. The favorable *ZmASB1* ^InDel+^ allele was enriched in modern maize inbred lines relative to teosinte and landraces. Together, these findings identify a natural cis‐regulatory mechanism controlling *ZmASB1* expression and support a *ZmASB1*‐tryptophan‐auxin module that contributes to root development and drought responses in maize seedlings.

## Graphical abstract

Natural variation in the maize *ZmASB1* promoter regulates tryptophan accumulation, root development, and drought tolerance through a tryptophan–auxin pathway. A 5‐bp InDel alters ZmDOF17 binding and promoter activity, and the favorable allele is enriched during breeding. These findings reveal a cis‐regulatory mechanism linking amino acid metabolism with hormone signaling and provide a target for drought‐resilient root improvement.

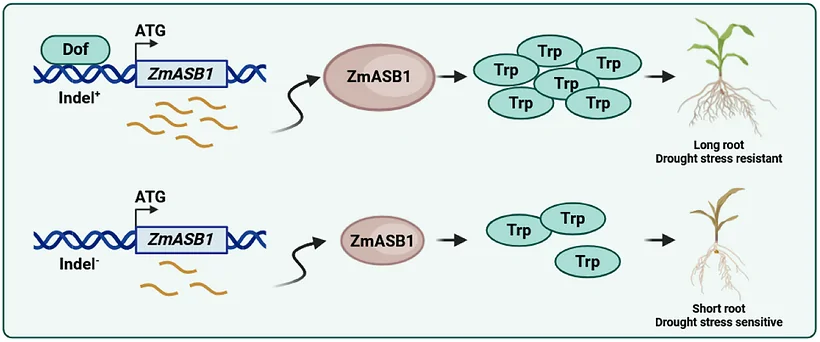

## 1 Introduction

Maize (*Zea mays* L.) is a globally important crop that supports food, feed, and industrial applications and therefore plays a crucial role in food security and agricultural economies [1, 2]. However, its production is increasingly threatened by abiotic stresses, particularly drought, as climate change intensifies and freshwater resources become more limited [3, 4]. This challenge highlights the need for drought‐tolerant maize varieties that produce high yields under water‐limited conditions. Root traits are important targets for improvement because roots play fundamental roles in water acquisition and plant survival under stress [5, 6]. A well‐developed root system with enhanced architecture, including increased root length, density, and depth, enables plants to take up water from a relatively large soil volume, thereby helping to alleviate the effects of drought [7, 8]. Furthermore, roots serve as the primary interface between plants and soil, mediating water uptake, nutrient acquisition, and signaling processes that coordinate whole‐plant responses to environmental stresses [9]. Elucidating the genetic and molecular mechanisms governing root development and identifying natural variations that enhance root traits are important steps toward developing climate‐resilient maize varieties.

Free amino acids (FAAs) contribute to plant growth, development, and stress responses [10]. They function as osmolytes for cellular osmotic adjustment, precursors for specialized metabolite biosynthesis, signaling molecules in various regulatory pathways, and nitrogen storage compounds [11, 12, 13]. FAA accumulation and metabolic flux are dynamically regulated by developmental cues and environmental stimuli, with specific amino acids playing particularly important roles in stress adaptation [14, 15]. As an essential aromatic amino acid, tryptophan (Trp) is a protein component and a precursor of secondary metabolites, including auxin, serotonin, and melatonin [14]. These metabolites contribute to seed germination, root growth and development, and senescence, as well as to responses to biotic and abiotic stresses [16, 17, 18]. Trp‐dependent auxin biosynthesis involves multiple enzymatic steps and regulatory checkpoints, and Trp conversion within this pathway contributes to the regulation of auxin homeostasis [19, 20]. Auxin regulates many aspects of root system architecture, including primary root elongation, lateral root initiation and emergence, root hair development, and gravitropism [21, 22]. The spatiotemporal distribution of auxin is established through coordinated regulation of its biosynthesis, transport, and degradation, generating concentration gradients that guide root developmental programs and adaptive responses to environmental cues [22, 23]. Therefore, genetic variation affecting Trp metabolism may influence auxin‐mediated root development and, ultimately, plant performance under drought.

Maize germplasm contains substantial genetic diversity accumulated during domestication and breeding, making it valuable for identifying beneficial alleles and clarifying the genetic architecture of complex traits [24]. Natural variation in metabolic profiles, including amino acid content, reflects adaptation to diverse environments and offers opportunities for crop improvement [25]. Traditional breeding approaches have exploited this diversity to increase yield and stress tolerance. However, metabolite profiles are internal biochemical traits that cannot be directly observed during routine phenotypic selection, limiting breeding efficiency. Genome‐wide association studies (GWAS) identify genetic loci associated with phenotypic variation at high resolution [24]. Metabolite‐based GWAS (mGWAS) can reveal the genetic basis of metabolic diversity and identify key genes/variants controlling metabolite accumulation, thereby facilitating molecular selection without direct metabolite quantification [26, 27]. Integrating GWAS with transcriptomics and metabolomics data can help construct regulatory networks and prioritize candidate genes within quantitative trait loci (QTLs) [28, 29]. Such integration can clarify relationships among genetic variants, gene expression, and metabolite abundance, thereby helping explain phenotypic variation [30].

In plants, Trp biosynthesis begins with the conversion of chorismate to anthranilate, catalyzed by anthranilate synthase (AS), the first committed and rate‐limiting enzyme in the pathway [15, 31]. AS is a heterodimer composed of α‐ and β‐subunits. The α‐subunit contains the catalytic domain and a C‐terminal regulatory domain harboring the Trp‐binding site that mediates feedback inhibition, whereas the β‐subunit provides glutamine amidotransferase activity [32, 33]. Given that AS functions as the gatekeeper of Trp biosynthesis, variation in the genes encoding AS subunits can affect Trp accumulation and downstream auxin production [15]. Despite progress in understanding amino acid metabolism and auxin‐mediated root development, the AS genes and regulatory mechanisms underlying natural variation in maize root Trp abundance, and how this variation relates to root architecture and drought responses, remain largely unexplored. Here, we integrated mGWAS, expression GWAS (eGWAS), gene‐expression correlations, haplotype analysis, promoter assays, and functional genetics to identify *ZmASB1* (GRMZM2G325131) as a regulator of root Trp abundance. We further characterized a naturally occurring 5‐bp promoter insertion/deletion (InDel) that affects *ZmDOF17*‐dependent promoter activity and proposed a working model linking *ZmASB1*‐dependent tryptophan metabolism with auxin‐associated root development and drought responses in maize seedlings.

## 2 Results

### 2.1 Natural Variation in Free Amino Acid Abundance in Maize Seedling Roots

To characterize natural variation in root amino acid abundance, we quantified 18 FAAs in seedling roots from 192 diverse maize inbred lines (Figure S1; Table S1): phenylalanine (Phe), methionine (Met), glutamic acid (Glu), arginine (Arg), lysine (Lys), tyrosine (Tyr), leucine (Leu), proline (Pro), Trp, serine (Ser), threonine (Thr), aspartic acid (Asp), valine (Val), isoleucine (Ile), histidine (His), alanine (Ala), glutamine (Gln), and asparagine (Asn). FAA abundance varied substantially among lines (Figure 1A), with coefficients of variation (CV) ranging from 16.74% to 91.18% (average CV = 38.10%). Broad‐sense heritability (*H* ^2^) ranged from 27.02% to 79.67%, with an average of 61.36% (Table S2). Correlation analysis revealed a structured association network among the 18 FAAs (Figure S2A), dominated by positive correlations. For example, the branched‐chain amino acids Val, Leu, and Ile formed a tightly correlated module (e.g., Val *vs* Ile, *r* = 0.77, *p* = 6.08 × 10^−5^), consistent with their shared biosynthetic pathways [34]. Phe and Tyr, both derived from the shikimate pathway, also showed strong co‐accumulation (*r* = 0.74, *p* = 4.51 × 10^−5^) [35]. We then examined relationships between the 18 root FAAs and 24 root traits (Figure S2B). Pro, Trp, His, Leu, Ser, Gln, Val, and Phe were positively correlated with root length, surface area, and volume traits, whereas the remaining ten FAAs showed weak negative correlations (*r* = −0.21 to −0.12); root diameter traits formed a separate cluster with an opposite correlation pattern.

**FIGURE 1:**
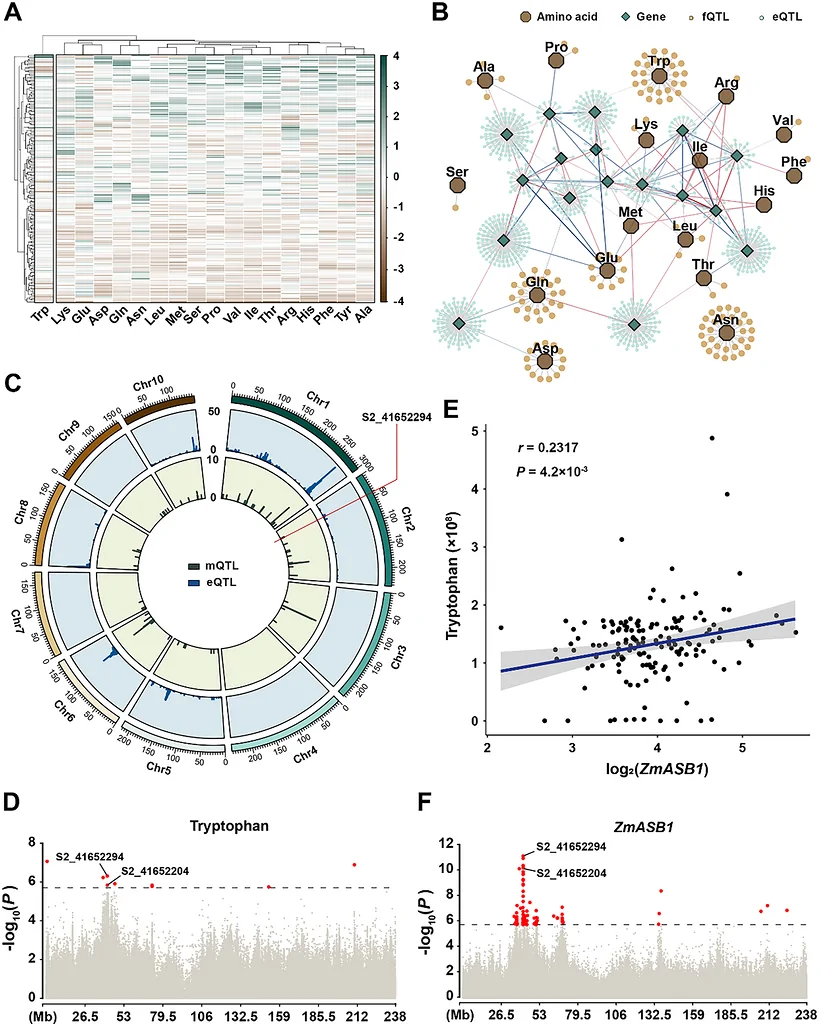
Natural variation in root free amino acid abundance and multi‐omics‐guided prioritization of *ZmASB1* in maize seedlings. (A) Hierarchical clustering heatmap of the abundances of 18 free amino acids (FAAs) across 192 maize inbred lines. Rows represent genotypes, and columns represent amino acids. For each genotype, root tissues from three seedlings were pooled to constitute one biological replicate, and two independent biological replicates were analyzed. Each cell represents the genotype‐level mean abundance, standardized as a z‐score for each amino acid across the 192 genotypes. Each biological sample was analyzed by a single LC‐MS injection. (B). Integrated network linking 18 FAAs, 17 candidate genes, 118 FAA‐associated quantitative trait loci (fQTLs), and 681 expression quantitative trait loci (eQTLs). Brown octagons, teal diamonds, orange circles, and light teal circles represent amino acids, candidate genes, fQTLs, and eQTLs, respectively. Amino acid‐fQTL and gene‐eQTL links were defined by significant metabolite‐based GWAS (mGWAS) and expression GWAS (eGWAS) associations, respectively, whereas amino acid‐gene links were retained for Pearson correlations satisfying |*r*| > 0.20 and *p* < 0.01. For amino acid‐gene correlation edges, red and blue indicate positive and negative correlations, respectively, and darker colors indicate larger absolute correlation coefficients. Edge thickness represents statistical significance, with thicker edges indicating smaller *p*‐values. The network was assembled and visualized using Cytoscape v3.10.2. (C) Circular plot showing the chromosomal distribution of FAA‐associated QTLs and eQTLs across the ten maize chromosomes. The inner and outer tracks represent FAA‐associated QTLs and eQTLs, respectively, and the radial scales indicate the number of associations in each track. Red connectors indicate colocalized FAA‐associated QTL‐eQTL loci. (D) Manhattan plot of the GWAS for root tryptophan abundance on chromosome 2. Each point represents one single‐nucleotide polymorphism (SNP); the *x*‐axis indicates physical position (Mb), and the *y*‐axis shows ‐log_10_(*p*). The dashed horizontal line indicates the genome‐wide significance threshold (*p* = 2.00 × 10^−6^). SNPs exceeding this threshold are highlighted in red, and the colocalized SNPs S2_41652294 and S2_41652204 are labeled. (E) Pearson correlation between *ZmASB1* transcript abundance and root tryptophan abundance across 192 maize inbred lines. Each point represents one inbred line based on genotype‐level mean values. The *x*‐axis shows log_2_‐transformed *ZmASB1* expression, and the *y*‐axis shows root tryptophan abundance. The blue line represents the fitted linear regression, and the gray shaded region indicates the 95% confidence interval. The Pearson correlation coefficient (*r*) and associated *p*‐value were calculated using a two‐sided Pearson correlation test. (F) Manhattan plot of the eGWAS for *ZmASB1* transcript abundance on chromosome 2. Each point represents one SNP; the *x*‐axis indicates physical position (Mb), and the *y*‐axis shows ‐log_10_(*p*). The dashed horizontal line indicates the genome‐wide significance threshold (*p* = 2.00 × 10^−6^). SNPs exceeding this threshold are highlighted in red, and the colocalized SNPs S2_41652294 and S2_41652204 are labeled.

### 2.2 Genome‐Wide Association Studies and Multi‐Omics Integration Analysis

To investigate the genetic basis of amino acid metabolism in maize seedling roots, we performed GWAS of the 18 FAAs in 192 inbred lines. This analysis used 13,361,037 high‐quality single‐nucleotide polymorphisms (SNPs) and the efficient mixed model association (EMMA). In total, 1,509 unique significant SNPs associated with the 18 FAAs were detected, with at least one significant association identified for each FAA (Figure S3; Figure S4). After linkage disequilibrium (LD) pruning, 118 FAA‐related QTLs (fQTLs) were identified (Figure S5A; Table S3). These results corresponded to approximately six fQTLs per FAA on average. Asn was associated with the largest number of QTLs, with 29 QTLs significantly associated with its variation, whereas no QTLs were detected for His, Ile, Met, or Tyr (Figure S5B). Based on lead SNPs and their LD intervals, a total of 274 fQTL‐associated genes were identified for the 118 fQTLs (Figure S5B). These candidate genes are involved in metabolic pathways including phenylalanine, tyrosine, and tryptophan biosynthesis, and amino acid biosynthesis (Table S4). For example, GRMZM5G826838 encodes glutamate decarboxylase (GAD), which has been reported to promote root growth and stress tolerance by modulating γ‐aminobutyric acid (GABA) biosynthesis in maize [36]. GRMZM2G328780 encodes a YUCCA family flavin monooxygenase involved in auxin biosynthesis [19], whereas GRMZM2G046966 and GRMZM2G325131 encode class I glutamine amidotransferase‐related proteins.

In our previous study [36, 37], root transcriptomes were generated for 357 maize inbred lines. Of these, 192 lines were selected for the present study, and their transcriptome data were used for FAA‐gene expression correlation and eGWAS analyses. Correlation analysis between the 18 FAAs and root gene expression levels revealed 16,016 significant FAA‐gene correlation pairs (*p* < 0.01, |*r*| > 0.2) (Figure S5C,D). The associated genes were involved in various metabolic pathways including secondary metabolite biosynthesis, amino acid biosynthesis, and plant hormone signaling (Table S5). To prioritize candidate genes underlying natural variation in amino acid abundance, we integrated these correlations with the fQTL results. A gene was retained if it was located within the fQTL interval associated with a specific amino acid and its expression was significantly correlated with that same amino acid. The intersection yielded 17 prioritized candidate genes involved in amino acid metabolism, including anthranilate synthases and glutamate decarboxylases, and cellular regulation, including a cyclin‐dependent kinase (Table S6).

We next performed eGWAS for 17 prioritized genes and identified 681 expression QTLs (eQTLs) (*p* < 2.00 × 10^−6^) (Figure S6; Table S7). Of these, 656 (96.33%) were trans‐eQTLs, and 25 were cis‐eQTLs. We constructed an FAA‐QTL‐gene network by integrating fQTLs, eQTLs, and FAA‐correlated genes (Figure 1B). The network comprised 18 amino acids, 17 candidate genes, 118 fQTLs, and 681 eQTLs. Notably, we identified a colocalized locus, S2_41652294, which was a strong eQTL for GRMZM2G325131 (*p* = 8.10 × 10^−12^) and was significantly associated with root Trp abundance (*p* = 4.92 × 10^−7^), suggesting that variation at this locus may influence Trp metabolism by modulating GRMZM2G325131 expression (Figure 1C).

### 2.3 *ZmASB1* Regulates Tryptophan Accumulation and Root Growth in Maize Seedlings

Trp is an essential aromatic amino acid involved in plant growth, development, and stress responses [38, 39, 40]. In our GWAS, Trp abundance was associated with 648 significant SNPs grouped into 27 fQTLs (Figure 1D). An fQTL at 41.65 Mb on chromosome 2 (S2_41652294, *p* = 4.92 × 10^−7^) contained, GRMZM2G325131, whose expression was significantly correlated with Trp abundance (*p* = 4.2 × 10^−3^, *r* = 0.2317) (Figure 1E). This gene encodes the β‐subunit of AS, which provides glutamine amidotransferase activity within the AS complex. Together with the α‐subunit, AS catalyzes the first committed step of the Trp biosynthesis pathway upstream of Trp‐dependent Indole‐3‐Acetic Acid (IAA) biosynthesis [32, 41, 42]. We therefore designated GRMZM2G325131 as *ZmASB1*. An eGWAS of *ZmASB1* expression identified 172 SNPs within its promoter region that were associated with its expression. Among these SNPs, S2_41652204 and S2_41652294 were colocalized with Trp fQTLs (Figure 1D,F; Figure S7A,B). These variants may cis‐regulate *ZmASB1* transcription, thereby influencing Trp biosynthesis.

To investigate the role of *ZmASB1* in Trp accumulation and root development, we obtained the Mutator insertional mutant (UFMu‐00576) from the UniformMu library (Figure S8). The transposon was inserted 609 bp upstream of the start codon (ATG) in the *ZmASB1* promoter region (Figure 2A,B). This mutant was designated as *zmasb1*. quantitative real‐time PCR (qRT‐PCR) showed that *ZmASB1* transcript levels were 24.02% lower in the *zmasb1* mutant than in wild‐type (WT) plants (*p* = 0.0184) (Figure 2C). Root Trp abundance was 42.87% lower in *zmasb1* than in WT (*p* = 0.0018) (Figure 2D). Total root length (TRL) in *zmasb1* was reduced by 11.20% (*p* = 0.0137) (Figure 2E,F), and root dry weight (RDW) was reduced by 7.26% (*p* = 0.0144) (Figure 2G). The *zmasb1* mutant also showed significant reductions in primary root length (PRL), lateral root length (LRL), and seminal root length (SRL), while shoot dry weight (SDW) was not significantly affected (Figure S9A–D).

**FIGURE 2:**
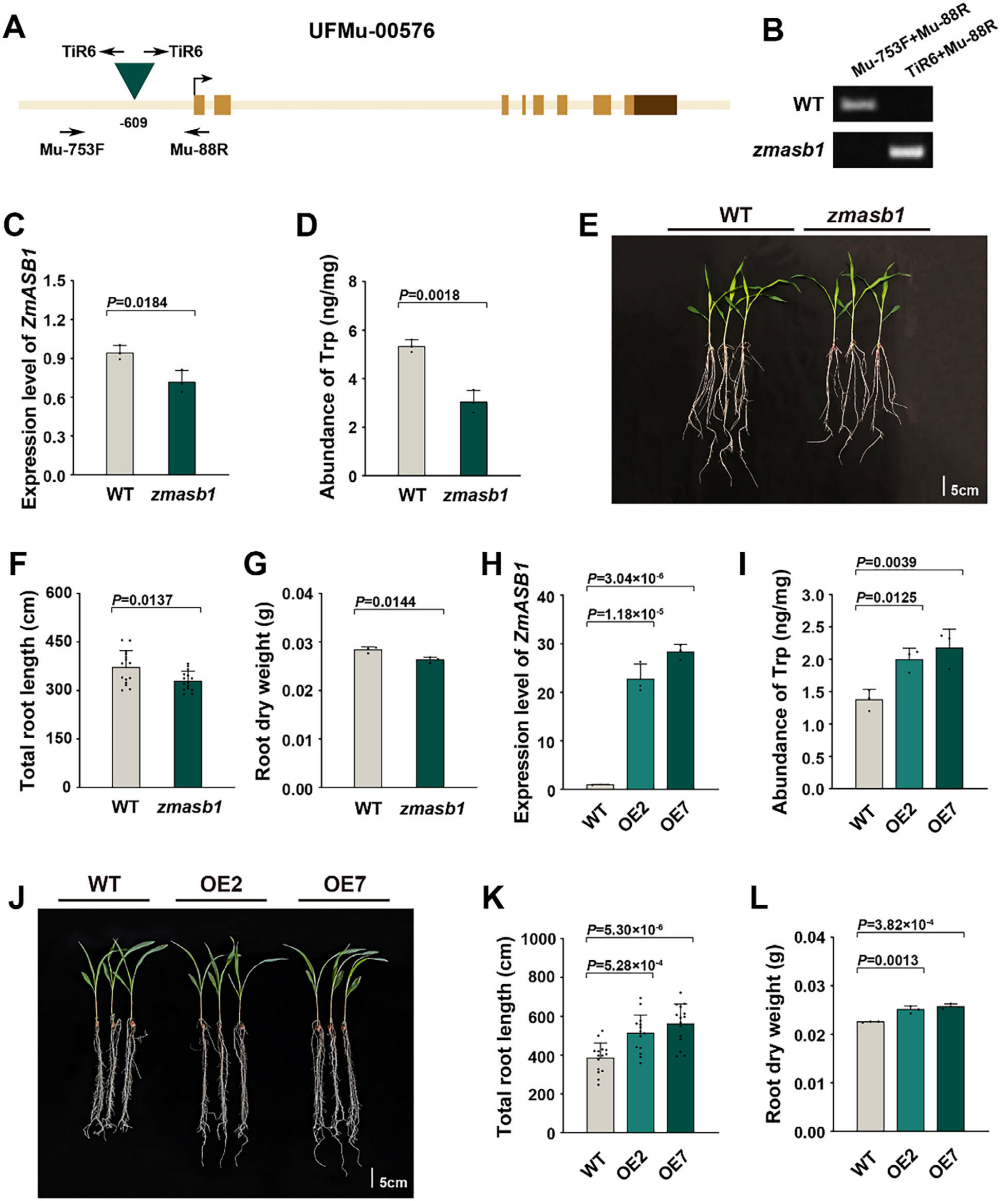
*ZmASB1* regulates tryptophan accumulation and root growth in maize. (A) Schematic representation of the *ZmASB1* gene structure and Mutator transposon insertion sites in *zmasb1* mutant. Green triangles indicate Mutator transposon insertions in the upstream promoter region of *ZmASB1*. Arrows indicate the orientation of the gene‐specific primers Mu‐753F and Mu‐88R and the degenerate transposon primer TiR6 used for genotyping. Gene structure and insertion sites were drawn based on the B73 reference genome annotation. (B) Representative agarose‐gel electrophoresis results for *zmasb1* genotyping. The Mu‐753F/Mu‐88R primer pair specifically amplifies the wild‐type (WT) allele, whereas the TiR6/Mu‐88R primer pair amplifies the mutant allele carrying the Mutator transposon insertion. (C, D) Quantification of *ZmASB1* expression level (**C**) and tryptophan abundance (**D**) in WT and *zmasb1* seedlings. (E) Representative images of WT (W22) and *zmasb1* seedlings. Scale bar = 5 cm. (F, G) Total root length (TRL; *n* = 15; **F**) and root dry weight (RDW; *n* = 3; **G**) in WT and *zmasb1* seedlings. (H, I) Quantification of *ZmASB1* expression level (**H**) and tryptophan abundance (I) in WT and OE seedlings. (J) Representative images of WT (B104) and OE seedlings. Scale bar = 5 cm. (K, L) TRL (*n* = 15; **K**) and RDW (*n* = 3; **L**) in WT and OE seedlings. Data are presented as mean ± standard deviation (SD). Statistical significance was determined by two‐sided Student's *t*‐tests (**C‐G**) and analysis of variance (ANOVA), followed by Fisher's least significant difference (LSD) test for post hoc pairwise comparisons (**H‐L**).

We generated two independent *ZmASB1*‐overexpression lines (OE2 and OE7 lines) in the B104 genetic background. qRT‐PCR showed that *ZmASB1* expression was 23.15‐ and 28.91‐fold higher in the OE2 and OE7 lines, respectively, than in WT plants (Figure 2H). Trp abundance was 45.27% and 58.58% higher in the two OE lines than in WT plants, respectively (Figure 2I). Both lines exhibited enhanced root growth (Figure 2J). TRL increased by 32.81% (*p* = 5.28 × 10^−4^) and 45.57% (*p* = 5.30 × 10^−6^) in the two lines, respectively (Figure 2K), while RDW increased by 11.24% (*p* = 0.0013) and 14.05% (*p* = 3.82 × 10^−4^) in the two OE lines, respectively (Figure 2L). In addition, both OE lines exhibited significant increases in PRL, LRL, SRL, and SDW (Figure S9E–H). These results indicate that *ZmASB1* positively regulates Trp abundance and root growth at the seedling stage.

### 2.4 *ZmASB1* Enhances Maize Seedling Drought Tolerance

A robust root system supports water uptake and drought tolerance [38, 44, 45]. To assess the role of *ZmASB1* in drought responses, we conducted soil‐based drought experiments with well‐watered (CK), drought (Dr), and re‐watering treatments in the *zmasb1* mutant and WT. Following drought and re‐watering, the survival rate of *zmasb1* was 14.44%, compared with 57.78% in WT (Figure 3A,B). The *zmasb1* mutant also showed reduced root growth and biomass under both CK and Dr (Figure 3C‐F; Figure S10A–D). Under Dr conditions, TRL decreased by 32.14% in *zmasb1* relative to WT, accompanied by reductions in PRL and LRL (Figure 3G; Figure S10B,C). RDW decreased by 36.41%, and SDW decreased by 33.68% (Figure 3H,I). Physiologically, water use efficiency (WUE) in *zmasb1* was reduced by 6.75% under CK and 8.73% under Dr relative to WT, and leaf relative water content (RWC) was reduced by 30.75% under Dr (Figure 3J,K). Stomatal conductance (*g*_s_) was also lower in *zmasb1*, whereas no significant differences were detected in stomatal density or aperture (Figure 3L; Figure S10E,F), suggesting that the changes in stomatal conductance were not associated with stomatal development. Root Trp abundance decreased by 28.30% and 33.09% under CK and Dr, respectively, while IAA abundance decreased by 54.05% and 62.66%, respectively (Figure 3M,N). To obtain independent support for these observations, we silenced *ZmASB1* using virus‐induced gene silencing (VIGS). Compared with empty‐vector controls, *ZmASB1*‐silenced plants showed reduced *ZmASB1* transcript levels, impaired root growth, lower biomass, and reduced survival after drought and re‐watering (Figure S11A–E). Root Trp and IAA abundances were also reduced in the silenced plants (Figure S11F,G).

**FIGURE 3:**
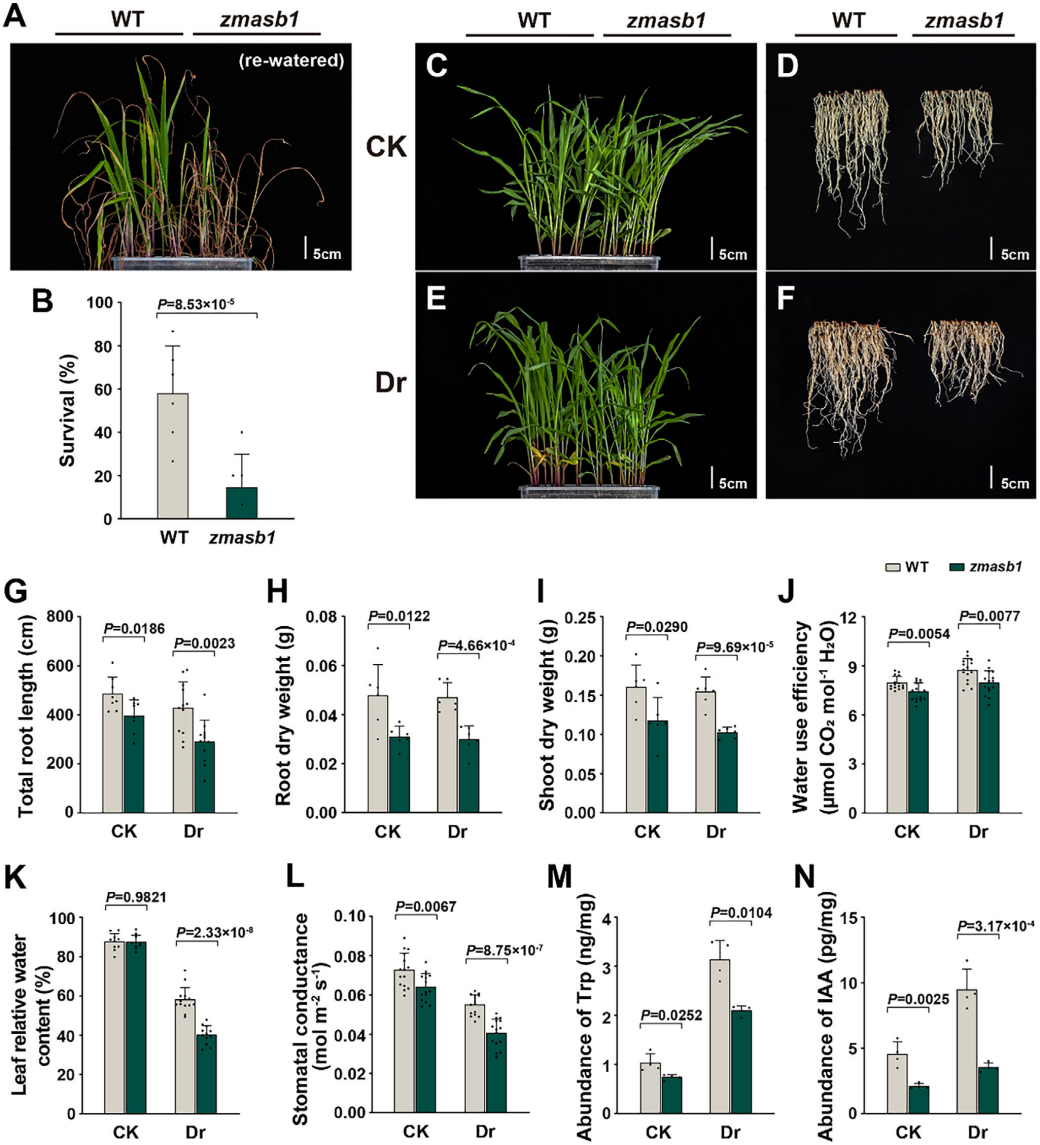
*ZmASB1* promotes drought tolerance in maize seedlings. (A) Representative images of WT (W22) and *zmasb1* mutant seedlings after drought stress followed by 3 days of re‐watering. Scale bar = 5 cm. (B) Survival rates of WT and *zmasb1* seedlings after drought stress and re‐watering. (C–F) Representative images of WT and *zmasb1* seedlings (C, E) and their root morphology (D, F) under well‐watered (CK) and drought (Dr) conditions at 14 days after planting. (G–L) Growth and physiological traits of WT and *zmasb1* seedlings under CK and Dr conditions, including TRL (G), RDW (H), shoot dry weight (SDW; I), water‐use efficiency (WUE; J), leaf relative water content (RWC; K), and stomatal conductance (*g*_s_; L). Sample sizes were *n* = 8 for CK and *n* = 12 for Dr in G, *n* = 6 in H‐I, *n* = 15 in J and L, and *n* = 10 in K. (M, N) Tryptophan (Trp; M) and indole‐3‐acetic acid (IAA; N) abundance in roots of WT and *zmasb1* seedlings under CK and Dr conditions (*n* = 4). Data are presented as mean ± SD. Statistical significance was determined by two‐sided Student's *t*‐test.

Both *ZmASB1* overexpression lines, OE2 and OE7, showed improved drought performance. Following drought and re‐watering, the survival rates of OE2 and OE7 were 44.44% and 53.33%, respectively, compared with 20.00% in WT plants (Figure 4A–C). Both lines maintained greater root growth and biomass than WT under CK and Dr conditions (Figure 4D–I; Figure S12A–D). Under Dr conditions, TRL increased by 22.08% in OE2 and 24.49% in OE7, while RDW increased by 21.15% and 23.30%, respectively (Figure 4J,K). SDW also increased by 12.71% and 16.37% in the two overexpression lines (Figure 4L). The overexpression lines also showed improved physiological performance. Under Dr conditions, WUE increased by 14.71% in OE2 and 9.19% in OE7 (Figure 4M), and both OE lines showed higher leaf RWC and *g*_s_ than WT (Figure 4N,O). Stomatal density and aperture did not differ significantly between the overexpression lines and WT (Figure S12E,F). Root Trp abundance was higher in both overexpression lines under CK and Dr conditions, with increases of 29.01% and 32.71% in OE2, and 33.40% and 30.71% in OE7, respectively (Figure 4P). Root IAA abundance also increased in both lines, particularly under Dr conditions (Figure 4Q). Together, these results indicate that *ZmASB1* is associated with root development, Trp and IAA abundance, and drought performance in maize seedlings.

**FIGURE 4:**
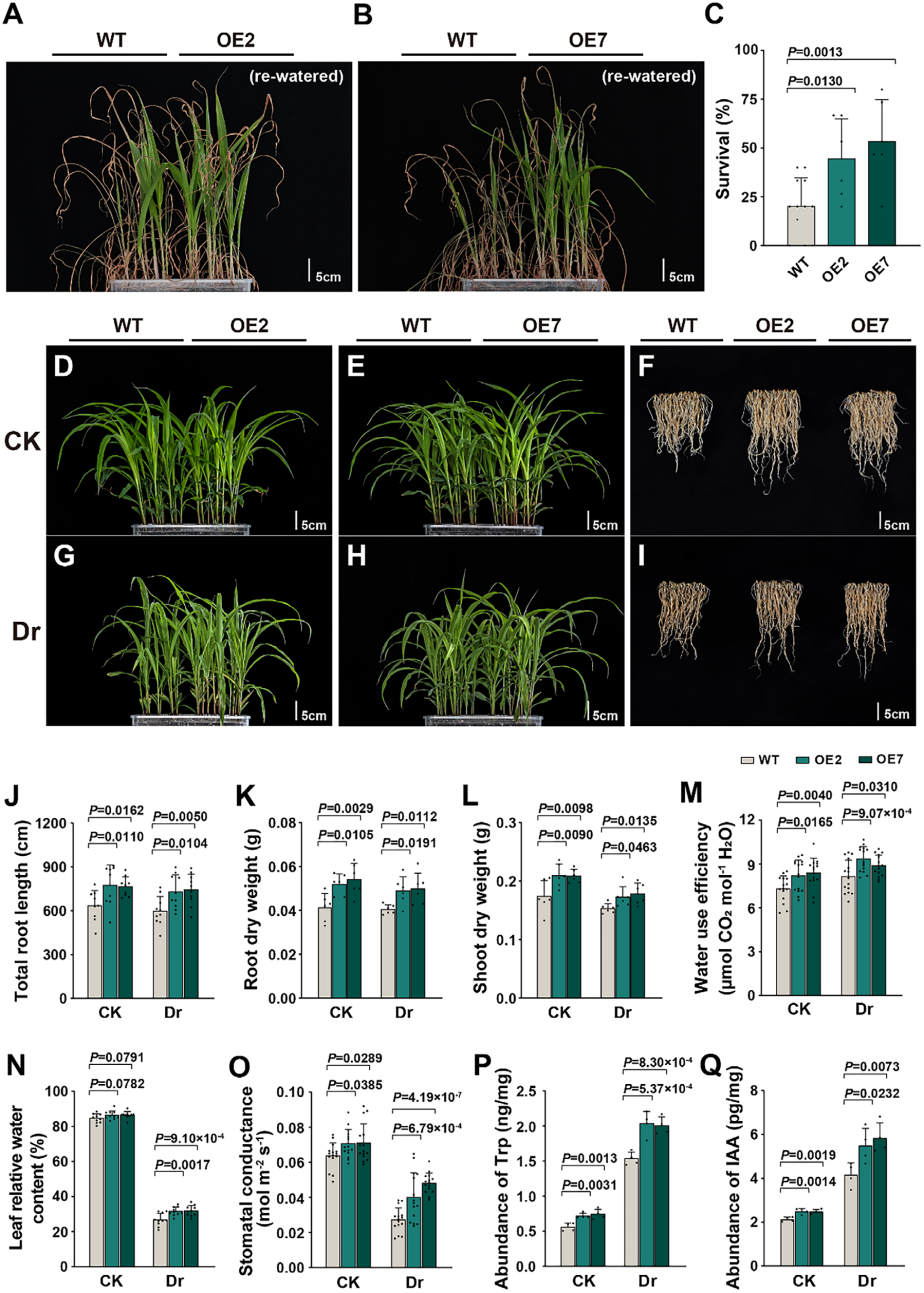
*ZmASB1* overexpression enhances drought tolerance in maize seedlings. (A, B) Representative images of OE2 (**A**) and OE7 (**B**) seedlings after drought stress following 3 days of re‐watering, with WT (B104) shown as control. Scale bar = 5 cm. (C) Survival rates of WT, OE2, and OE7 seedlings after drought stress and re‐watering (*n* = 6). (D–I) Representative images of WT, OE2, and OE7 seedlings (**D**, **E**, **G**, and **H**) and their root morphology (**F** and **I**) under CK (**D**‐**F**) and Dr (**G**‐**I**) conditions at 14 days after planting. (J–O) Growth and physiological traits of WT, OE2, and OE7 seedlings under CK and Dr conditions, including TRL (**J**), RDW (**K**), SDW (**L**), WUE (**M**), RWC (**N**), and *g*_s_ (**O**). Sample sizes were *n* = 9 for CK and *n* = 10 for Dr in J, *n* = 6 in K‐L, *n* = 15 in M and O, and *n* = 10 in N. (P, Q) Trp (**P**) and IAA (**Q**) abundance in roots of WT, OE2, and OE7 seedlings under CK and Dr conditions (*n* = 4). Data are presented as mean ± SD. Statistical significance was determined by ANOVA, followed by LSD test for post hoc pairwise comparisons.

### 2.5 *ZmASB1* Enhances Root Growth and Drought Tolerance Through Tryptophan‐Dependent Auxin Biosynthesis

To investigate molecular processes associated with *ZmASB1*‐dependent root and drought phenotypes, we performed RNA sequencing (RNA‐seq) on roots from *zmasb1*, OE2, OE7, and their corresponding WT controls under CK and Dr conditions. Both loss and overexpression of *ZmASB1* caused substantial transcriptional reprogramming (Figure S13A,B). We focused on differentially expressed genes (DEGs) showing opposite expression patterns in *zmasb1* and the overexpression lines. Kyoto Encyclopedia of Genes and Genomes (KEGG) analysis identified pathways related to amino acid metabolism, including biosynthesis of amino acids and phenylalanine, tyrosine, and tryptophan biosynthesis, as well as plant hormone signal transduction, phenylpropanoid and glutathione metabolism, secondary metabolite biosynthesis, nitrogen metabolism, ABC transporters, and MAPK signaling (Figure S13C; Table S8). These enrichments were consistent with a role for *ZmASB1* in Trp‐IAA metabolism and drought responses, and further highlighted related changes in interconnected metabolic and stress‐response pathways. We examined genes involved in Trp biosynthesis and downstream IAA production. Several genes showed expression patterns consistent with *ZmASB1* activity. These genes included GRMZM2G119881, encoding another anthranilate synthase β‐subunit, and the tryptophan synthase β‐subunit genes GRMZM2G005024 (*orp2*) and GRMZM2G169593 (*orp1*) (Figure S13D). In addition, the putative IAA‐associated genes GRMZM2G111309 and GRMZM2G443495 were upregulated in the overexpression lines under drought conditions (Figure S13D).

To test whether reduced Trp and IAA availability contributes to the *zmasb1* root‐growth defect, we supplied Trp or IAA to mutant seedlings under hydroponic conditions. PRL was 12.42% lower in *zmasb1* than in WT plants (*p* = 5.99 × 10^−9^; Figure S14A). Supplementation with 10 µM Trp partially restored primary root length, with an increase of 5.87% (*p* = 0.0048). Similarly, 0.1, 0.5 and 2.5 nM IAA increased PRL by 3.98% (*p* = 0.0303), 6.57% (*p* = 5.94 × 10^−4^) and 5.23% (*p* = 0.0052), respectively (Figure S14B). These treatments therefore partially alleviated the root‐growth defect of *zmasb1*.

We next evaluated whether Trp or IAA application could improve the drought‐sensitive phenotype of *zmasb1*. WT and *zmasb1* seedlings were treated with 0.1% Tween (mock), 100 µM Trp, or 10 µM IAA before drought treatment. Following drought and re‐watering, the survival rate of mock‐treated *zmasb1* seedlings was 11.11%, compared with 61.11% for mock‐treated WT seedlings (Figure 5A,B). Trp and IAA increased mutant survival to 47.22% and 58.33%, respectively. Both treatments also improved root and shoot growth under CK and Dr conditions (Figure 5C–F; Figure S15A–D). Under drought, TRL increased by 29.05% after Trp treatment and by 27.30% after IAA treatment (Figure 5G), while RDW increased by 39.24% and 42.00%, respectively (Figure 5H). SDW also increased by 20.91% and 16.03%, respectively (Figure 5I). Both treatments also improved WUE, RWC, and *g*_s_ under drought (Figure 5J–L). Root Trp and IAA abundance increased after both treatments, particularly under drought conditions (Figure 5M,N). Together, the transcriptomic and rescue analyses support the involvement of Trp‐IAA metabolism in *ZmASB1*‐associated root growth and drought responses.

**FIGURE 5:**
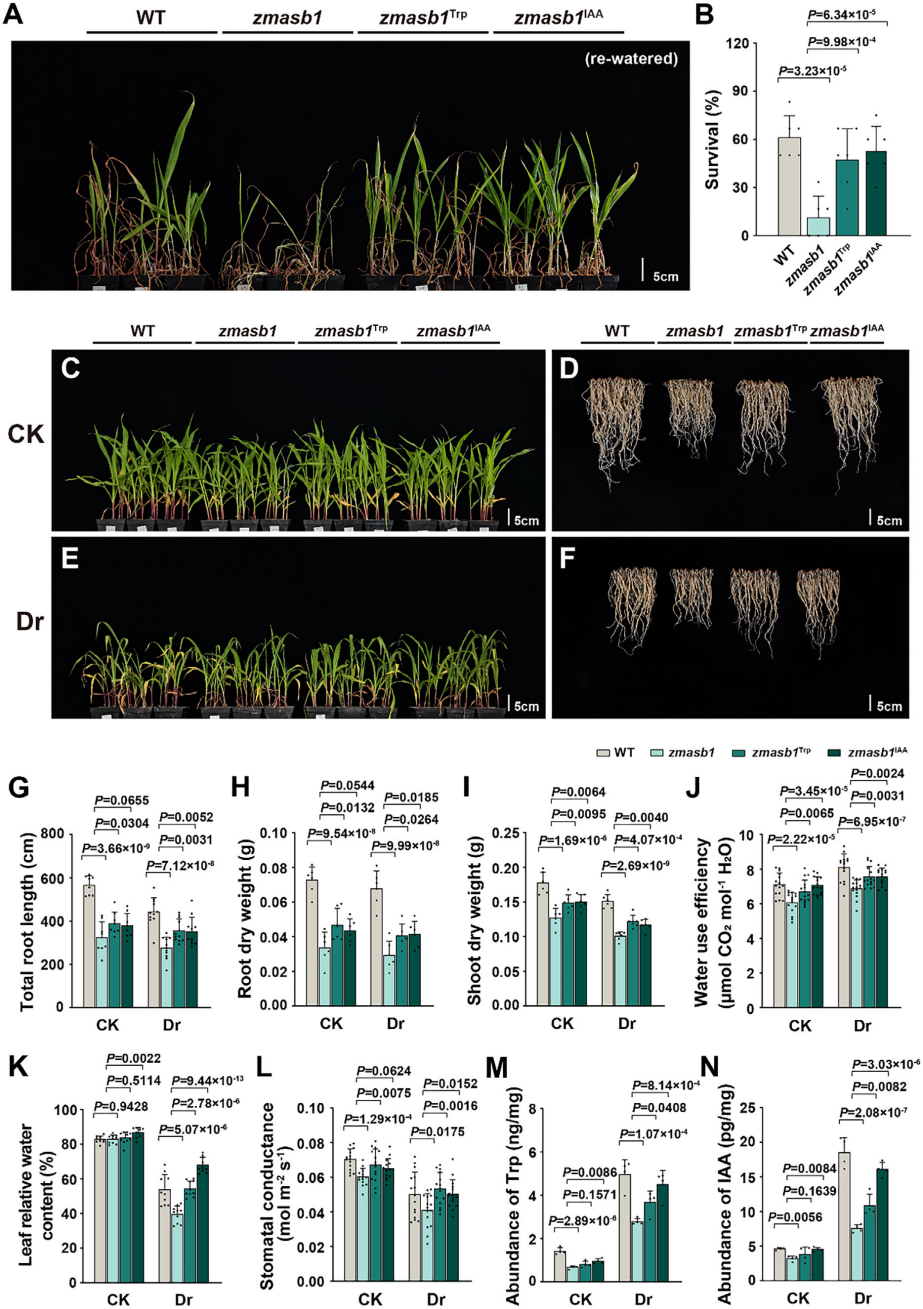
Exogenous application of Trp or IAA partially alleviates the drought‐sensitive phenotype of *zmasb1* mutant. (A) Representative images of WT, *zmasb1*, *zmasb1* treated with 100 µM Trp (*zmasb1* ^Trp^), and *zmasb1* treated with 10 µM IAA (*zmasb1* ^IAA^) after drought stress following 3 days of re‐watering. Trp‐ and IAA‐treated plants were sprayed with 100 µM Trp or 10 µM IAA, respectively, in 0.1% Tween solution; control plants were sprayed with 0.1% Tween solution alone. Scale bar = 5 cm. (B) Survival rates of WT, *zmasb1*, *zmasb1* ^Trp^, and *zmasb1* ^IAA^ seedlings after drought stress and re‐watering (*n* = 6). (C–F) Representative images of WT, *zmasb1*, *zmasb1* ^Trp^, and *zmasb1* ^IAA^ seedlings under CK (**C** and **D**) and Dr (**E** and **F**) conditions at 14 days after planting. (G–L) Growth and physiological traits of WT, *zmasb1*, *zmasb1* ^Trp^, and *zmasb1* ^IAA^ seedlings under CK and Dr conditions, including TRL (**G**), RDW (**H**), SDW (**I**), WUE (**J**), RWC (**K**), and *g*_s_ (**L**). Sample sizes were *n* = 8 and 11 for CK and Dr in G, respectively; *n* = 6 in H‐I; *n* = 15 in J; *n* = 10 in K; and *n* = 15 in L. (M, N) Trp (**M**) and IAA (**N**) abundance in roots of WT, *zmasb1*, *zmasb1* ^Trp^, and *zmasb1* ^IAA^ seedlings under CK and Dr conditions (*n* = 4). All data are presented as mean ± SD. Statistical significance was determined by ANOVA, followed by LSD test for post hoc pairwise comparisons.

### 2.6 Natural Variation in the *ZmASB1* Promoter Affects the Binding Affinity for ZmDOF17

To investigate the genetic basis of variation in Trp abundance and *ZmASB1* expression, we resequenced *ZmASB1* in 169 inbred lines. The analysis covered a 7,543‐bp region spanning the promoter, coding, and downstream regions. In total, 385 variants were identified (284 SNPs and 101 InDels with a minor allele frequency (MAF) > 0.05). A *ZmASB1*‐based association analysis identified 93 variants significantly associated with *ZmASB1* expression, including 62 SNPs and 28 InDels in the promoter region (Figure 6A). Of these, 16 (nine SNPs and seven InDels) were also significantly associated with Trp abundance (Figure 6B). These variants showed strong pairwise LD (Figure 6C). Based on these variants, the inbred lines were classified into four major haplotypes (Figure 6D). Compared with Hap4, Hap1 showed 70.62% higher root Trp abundance (*p* = 1.14 × 10^−5^) and 67.59% higher *ZmASB1* expression (*p* = 1.95 × 10^−14^) (Figure 6E,F). We cloned a 758‐bp promoter fragment upstream of ATG from Hap1 and Hap4, which covered the above‐mentioned 16 variant sites, and tested their activities using a dual‐luciferase reporter assay in *Nicotiana benthamiana* leaves. The Hap1 promoter showed 1.90‐fold higher relative luciferase activity than the Hap4 promoter (*p* = 0.0031) (Figure 6G). These results suggest that promoter sequence variation contributes to differences in *ZmASB1* expression.

**FIGURE 6:**
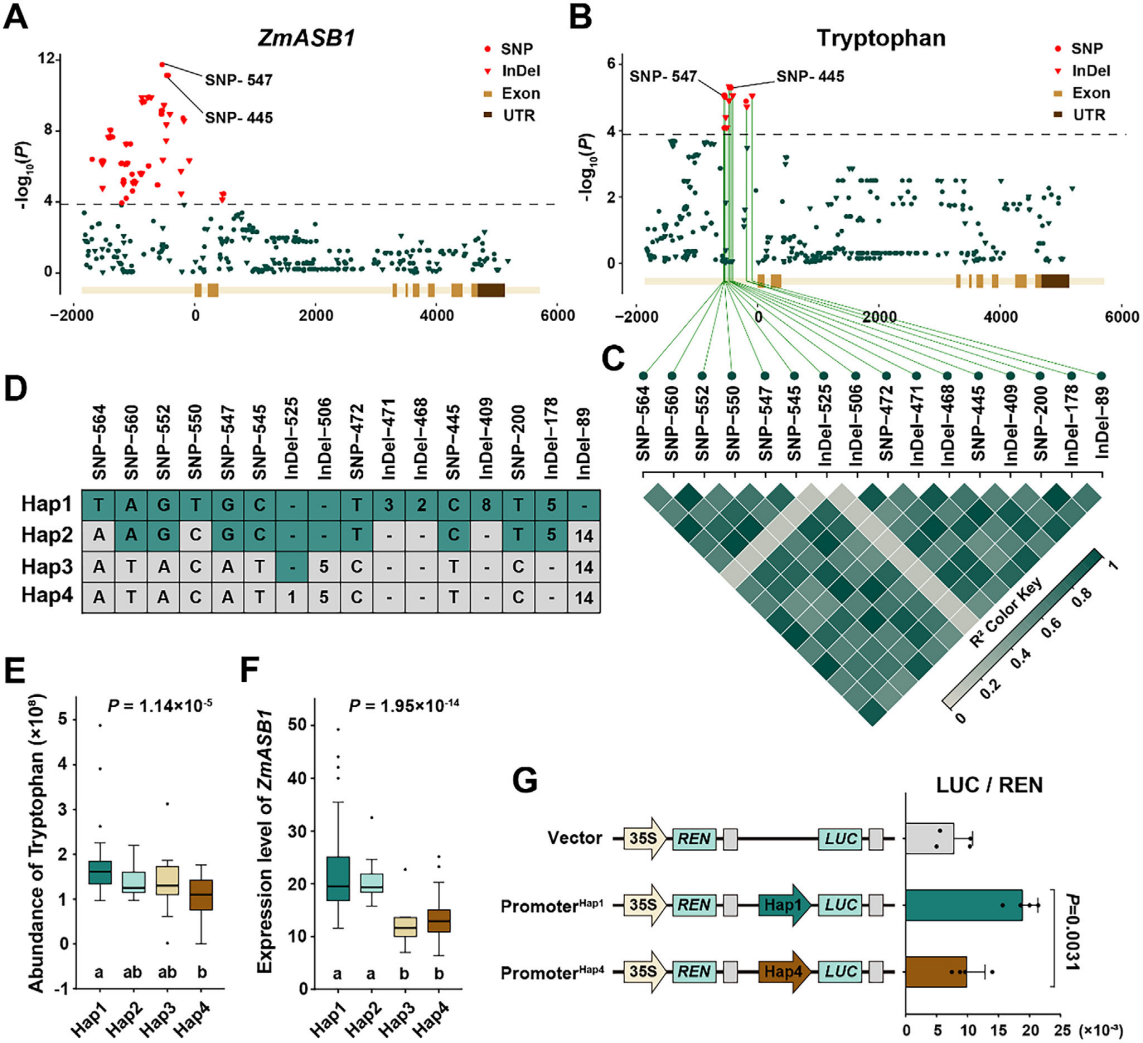
Natural variation in the *ZmASB1* promoter modulates its expression and tryptophan accumulation. (A, B) Regional association plots for *ZmASB1* expression (**A**) and tryptophan abundance (**B**). The *x*‐axis represents position relative to the *ZmASB1* start codon (bp), and the *y*‐axis represents ‐log_10_(*p*). The dashed horizontal line indicates the significance threshold (‐log_10_(*p*) > 3.89). The *ZmASB1* gene model is shown below each plot. Circles and triangles represent SNPs and insertions/deletions (InDels), respectively; significant variants are highlighted in red. Orange and brown rectangles indicate exons and untranslated regions(UTRs), respectively. (C) Pairwise linkage disequilibrium (LD) among the 16 promoter variants associated with both *ZmASB1* expression and root tryptophan abundance. Color intensity indicates pairwise *r* ^2^, ranging from 0 (light gray) to 1 (dark green). (D) Four major promoter haplotypes (Hap1‐Hap4) defined by the 16 variants. (E, F) Boxplots of tryptophan abundance (**E**) and *ZmASB1* expression (**F**) for the four haplotype groups. Differences among haplotypes were assessed using ANOVA followed by LSD test. (G) Dual‐luciferase reporter assay comparing the activities of the Hap1 and Hap4 promoters. Reporter constructs are shown on the left, with the empty reporter vector included as a control. Data are presented as mean ± SD (*n* = 4). The Hap1 and Hap4 promoters were compared using a two‐sided Student's *t*‐test.

We predicted transcription factor‐binding sites in the 758‐bp *ZmASB1* promoter using PlantPAN3.0. InDel‐178, which represents the presence or absence of a 5‐bp sequence “AACTT”, overlapped a predicted DNA‐binding with one finger (DOF) transcription factor‐binding motif (Figure 7A). We therefore tested whether InDel‐178 explained the activity difference between the Hap1 and Hap4 promoter haplotypes. Three pairs of matched promoter fragments were analyzed using dual‐luciferase assays: one fragment containing the upstream promoter variants, one differing only at InDel‐178, and one containing the downstream variant. The upstream and downstream fragment pairs showed no significant difference in reporter activity between Hap1 and Hap4. In contrast, the fragment carrying the InDel^+^ allele showed significantly higher activity than the corresponding InDel^−^ fragment (Figure 7B). InDel‐178 is therefore a leading candidate functional variant underlying the difference in *ZmASB1* promoter activity between the two haplotypes. Co‐expression analysis of *ZmASB1* and DOF transcription factor genes was performed using root transcriptomes of 192 inbred lines. This analysis identified 18 DOF genes whose expression levels were significantly correlated with *ZmASB1* expression (Figure 7C). Among these genes, *ZmDOF17* showed the strongest correlation with *ZmASB1* expression (*r* = 0.3461, *p* = 2.04 × 10^−5^). Consistent with the co‐expression analysis, published ChIP‐seq data [45] indicated that the *ZmASB1* promoter region containing InDel‐178 is occupied by ZmDOF17, ZmDOF21, ZmDOF22, and ZmDOF30 (Figure 7D). To test whether ZmDOF17 differentially regulates the two InDel‐178 alleles, we performed dual‐luciferase assays using promoter fragments containing only the InDel‐178 region. In the absence of ZmDOF17, the Hap1 fragment showed higher reporter activity than the Hap4 fragment (*p* = 0.0067; Figure 7E,F). Co‐expression with ZmDOF17 increased the activity of the Hap1 reporter by 7.69‐fold (*p* = 2.09 × 10^−^^4^), whereas no comparable activation was detected for the Hap4 reporter. Electrophoretic mobility shift assay (EMSA) further showed that ZmDOF17 bound to both promoter probes, but the binding signal was stronger for the Hap1 probe than for the Hap4 probe (Figure 7G). These results support a model in which InDel‐178 may alter ZmDOF17 binding and thereby contribute to differences in *ZmASB1* promoter activity.

**FIGURE 7:**
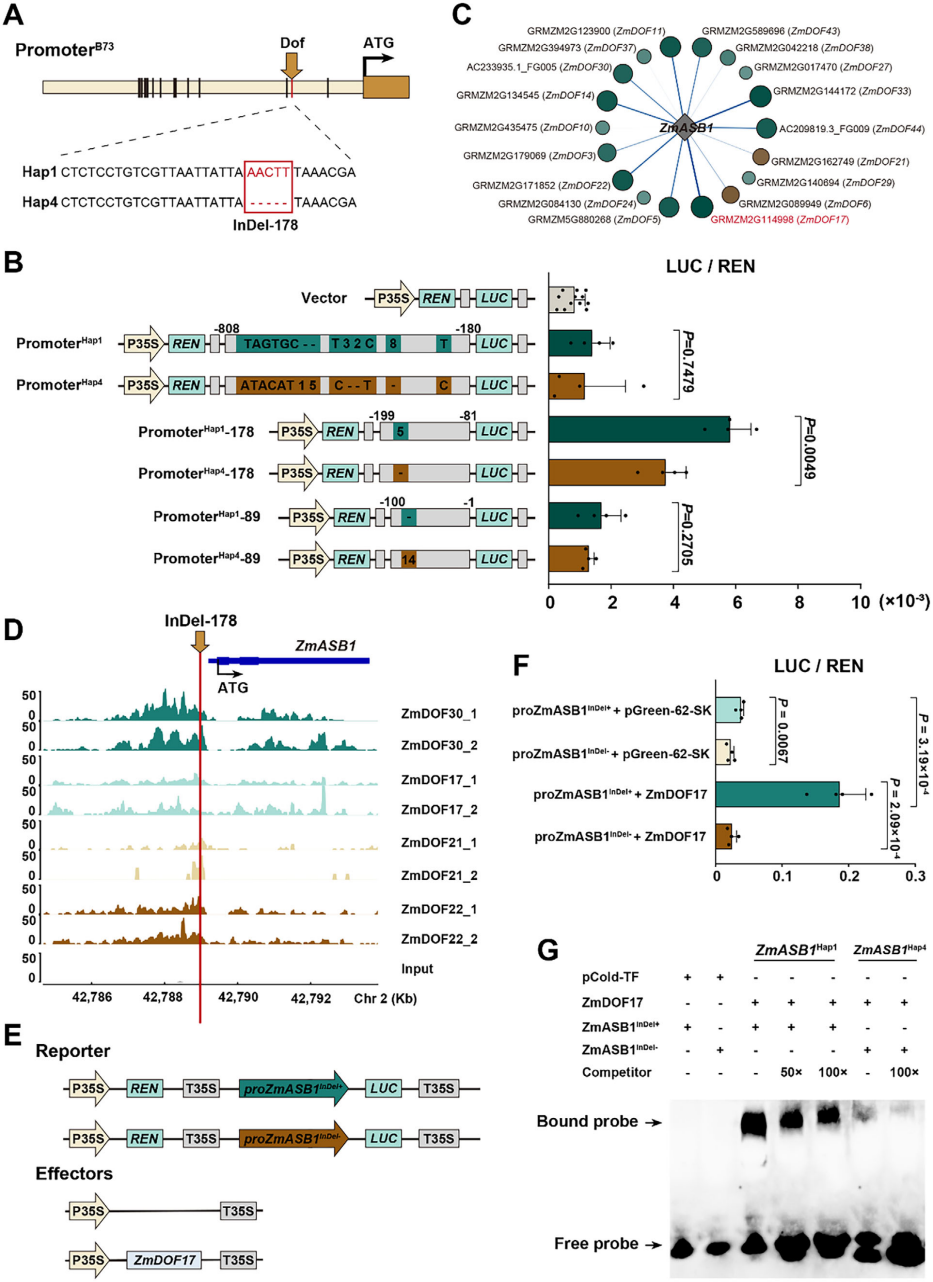
The InDel‐178 is a leading functional variant affecting DOF transcription factor binding and *ZmASB1* promoter activity. (A) Schematic of the *ZmASB1* promoter showing the position of InDel‐178 within a predicted DOF transcription factor binding site. The sequence alignment shows the 5‐bp insertion (“AACTT”) present in Hap1 but absent in Hap4. (B) Segmented dual‐luciferase analysis of the *ZmASB1* promoter. The promoter was divided into three segments: the upstream fragment (‐808 to ‐180), a fragment containing InDel‐178 (‐199 to ‐81), and a downstream fragment (‐100 to ‐1). Hap1 and Hap4 constructs were generated for each segment. The left panel shows the reporter constructs, and the right panel shows the corresponding relative luciferase activity (LUC/REN). Data are presented as mean ± SD (*n* = 4). Statistical significance was assessed using two‐sided Student's *t*‐tests. (C) Co‐expression network of *ZmASB1* and associated DOF‐family transcription factors in a maize association panel (*n* = 192). Node color indicates the direction and strength of the correlation, with green and brown representing positive and negative correlations, respectively. Node size is proportional to statistical significance. (D) Public ChIP‐seq profiles showing the occupancy of ZmDOF proteins at the ZmASB1 promoter. (E) Schematic diagrams of the reporter and effector constructs used in the dual‐luciferase assay. (F) Dual‐luciferase assay measuring the transcriptional activation of *ZmASB1* promoter variants by ZmDOF17. Data are presented as mean ± SD (*n* = 4). Statistical significance was determined by two‐sided Student's *t*‐test. (G) Electrophoretic mobility shift assay showing the binding of ZmDOF17 to the InDel^+^ and InDel^−^ promoter probes. The shifted bands indicate probe‐protein complexes.

To obtain independent support for the relationship between *ZmDOF17* and *ZmASB1*, we performed VIGS of *ZmDOF17* in maize seedlings. Silencing *ZmDOF17* reduced *ZmASB1* transcript levels relative to empty‐vector controls (Figure S16A,B). Silenced plants also showed reduced drought performance, with survival rate decreased by 14.66% (Figure S16C). Under CK and Dr conditions, TRL decreased by 21.98% and 17.98%, respectively, whereas RDW decreased by 31.90% and 28.95%, respectively (Figure S16D–F). Root Trp and IAA abundances were also significantly reduced in *ZmDOF17*‐silenced plants compared with empty‐vector controls (Figure S16G,H). These results independently support a role for *ZmDOF17* in *ZmASB1*‐associated root development and drought responses.

Finally, we examined the allele frequencies of InDel‐178 across maize germplasm. The InDel^+^ allele was present at a low frequency in teosinte (10.84%; *n* = 83) and landraces (12.68%; *n* = 71), but was more frequent in modern inbred lines (46.29%; *n* = 350) (Figure 8A; Table S10). In Chinese inbred lines, the frequency of InDel^+^ increased from 50.59% in lines released before 2000 (*n* = 85) to 67.92% in lines released after 2000 (*n* = 159) (Figure 8B; Table S10).

**FIGURE 8:**
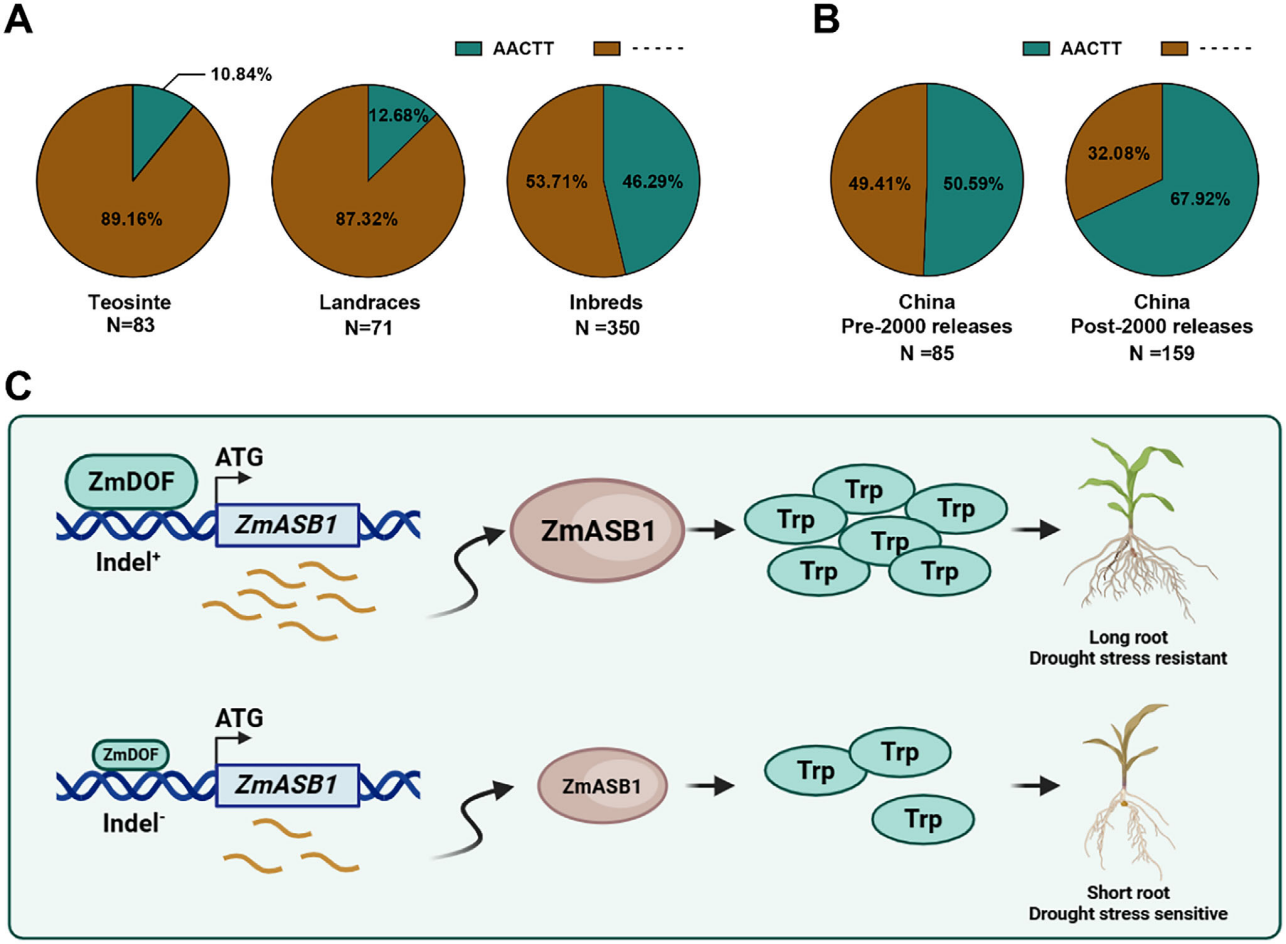
Allele frequency distribution of InDel‐178 across maize germplasm and a proposed working model for *ZmASB1*‐mediated root growth and drought tolerance. (A) The allele frequency of InDel‐178 in teosinte (*n* = 83), landraces (*n* = 71), and inbred lines(*n* = 350). (B) Allele frequencies of InDel‐178 in Chinese maize inbred lines released before 2000 (*n* = 85) and after 2000 (*n* = 159). (C) A proposed working model for how the InDel‐178 variant in the *ZmASB1* promoter regulates root growth and drought tolerance. The presence of the favorable 5‐bp insertion (InDel^+^) enhances the binding of a DOF transcription factor, leading to higher *ZmASB1* expression. This boosts Trp biosynthesis, which in turn promotes robust root development and is associated with improved drought performance. Conversely, the allele lacking the insertion (InDel^−^) results in lower *ZmASB1* expression, reduced Trp levels, compromised root growth, and drought sensitivity.

## 3 Discussion

Increasing drought frequency and severity highlight the need for climate‐resilient maize varieties with robust root systems for efficient water uptake [46]. We investigated the genetic basis of natural variation in maize root amino acid profiles and identified *ZmASB1*, an AS β‐subunit, as a regulator associated with root Trp accumulation. Our data support a role for *ZmASB1* in root growth and drought performance in association with Trp‐dependent auxin biosynthesis. We also identified a promoter InDel‐178 that affects promoter activity and ZmDOF17 binding. The favorable *ZmASB1* alleles were enriched in modern maize inbred lines. These findings clarify the intricate regulatory mechanisms linking amino acid metabolism, hormone signaling, and stress adaptation in maize, while providing insights into valuable genetic resources relevant to the development climate‐resilient varieties.

FAAs play diverse roles in plant responses to abiotic stress, and their levels often increase under stress conditions [47]. In our panel, the abundances of 18 FAAs varied widely among maize roots (Figure 1A). This variation exceeded that reported for amino acids in maize kernels [48] and was comparable to that in rice and wheat grains [49, 50]. The relatively high broad‐sense heritability further indicates the strong genetic control of root amino acid metabolism and its potential for genetic improvement [48, 49, 50]. Using 13,361,037 high‐quality SNPs from whole‐genome resequencing, we identified 1,509 significant SNPs associated with the 18 FAAs and assigned them to 118 fQTLs. Compared with our previous root‐metabolome GWAS, which used 300,877 transcriptome‐derived SNPs and detected 108 significant SNPs within 17 QTLs [36], the current analysis provided broader genomic coverage and identified a larger number of associated loci. Integrating GWAS and transcriptomic data further prioritized 17 candidate genes whose expression were significantly correlated with the corresponding amino acid traits and whose genomic regions were supported by genetic associations. Consistent with its applications in other crops [30, 51, 52], this integrative approach narrowed the set of candidate genes and provided a basis for subsequent functional characterization.

Trp biosynthesis proceeds from chorismate through a series of reactions involving anthranilate synthase, phosphoribosyl anthranilate transferase, phosphoribosyl anthranilate isomerase, indole‐3‐glycerol phosphate synthase, and tryptophan synthase [53, 54, 55]. Functional analyses indicated that *ZmASB1* acts as a positive regulator of Trp biosynthesis, consistent with its role as the β‐subunit of anthranilate synthase [56, 57]. Changes *ZmASB1* expression altered root Trp and IAA contents, thereby affecting maize root growth. Exogenous Trp and IAA partially restored root length, supporting the possibility that reduced Trp and IAA availability contributes to the abnormal *zmasb1* root phenotype. This finding is consistent with previous work in rice, and with conserved role of the Trp‐auxin pathway in regulating root growth across cereal crops [57]. Trp metabolism has been implicated in plant responses to drought, salinity, and heavy metals [25, 58, 59]. Under drought stress, plants tend to accumulate Trp to enhance their resistance to stress [25]. Foliar application of Trp can alleviate the adverse effects of drought in maize, supporting a role for Trp in drought tolerance [25]. In our study, *ZmASB1* overexpression was associated with enhanced root growth and drought tolerance, whereas Trp application improved survival and root performance in drought‐stressed *zmasb1* plants. These findings support a link between *ZmASB1*‐dependent root‐system architecture and drought performance. Trp may contribute to drought adaptation through Trp‐dependent auxin biosynthesis, thereby promoting root system development, which may improve soil exploration and help maintain plant water status under drought [43, 44]. Consistent with this possibility, the higher shoot biomass, leaf RWC, *g*_s_, and WUE observed in the overexpression lines may partly reflect improved plant water status associated with their more developed root systems. Intrinsic root hydraulic properties remain to be determined. Beyond Trp‐IAA metabolism, transcriptomic enrichment also suggests possible contributions from nitrogen, glutathione, phenylpropanoid, and carbohydrate metabolism to the *ZmASB1*‐associated drought response, but these pathways remain to be validated (Figure S13C).

Natural variation in *ZmASB1* may help explain the diversity in Trp levels and root growth. Variation in promoter influences gene expression by altering transcription factor binding, chromatin accessibility, and chromatin structure [60, 61]. We identified InDel‐178, a 5‐bp AACTT insertion/deletion, as the leading candidate functional variant underlying differences in promoter activity. This variant alters a putative DOF‐binding motif, suggesting a possible effect on transcriptional regulation. DOF proteins are plant‐specific transcription factors involved in plant growth, flowering, fruit ripening, and responses to biotic and abiotic stresses [62]. They recognize AAAG or CTTT motifs in the promoter region of their target genes [63]. InDel‐178 overlaps a putative DOF transcription factor‐binding motif, and our EMSA and dual‐luciferase assays showed that the InDel^+^ allele, which contains the 5‐bp AACTT insertion, has a higher affinity for ZmDOF17 and stronger ZmDOF17‐dependent promoter activity than the InDel^−^ allele. This allele‐dependent transcriptional regulation provides a plausible molecular link between promoter sequence variation and the differences in *ZmASB1* expression, root Trp abundance, and root growth observed between haplotypes. Nevertheless, natural haplotypes contain additional linked variants, allele‐specific editing or near‐isogenic materials will be needed to resolve the allele‐specific contribution. Population genetic analyses showed that the favorable *ZmASB1* ^InDel+^ allele was enriched in modern maize inbred lines. Its frequency was low and similar in teosinte and landraces but substantially higher in modern inbred lines, particularly in more recently released Chinese inbred lines (Figure 8A,B). This pattern is consistent with enrichment of alleles favored during crop improvement [64, 65]. The enrichment of *ZmASB1* ^InDel+^ in modern germplasm suggests that enhanced Trp metabolism, auxin‐mediated root growth, and drought tolerance may have been unintended but advantageous consequences of maize breeding efforts aimed at improving overall adaptation and productivity. Taken together, our integrated natural‐variation, promoter, biochemical, and functional analyses support a regulatory role for *ZmASB1* in root tryptophan metabolism, auxin‐associated root development, and drought responses, while further suggesting that InDel‐178 contributes to variation in this regulatory module. Extending these analyses beyond the seedling stage and to field‐relevant environments could help determine the consistency and agronomic relevance of the observed effects, particularly given the polygenic and environment‐dependent nature of maize drought tolerance.

In summary, this study identifies *ZmASB1* as a genetic factor associated with natural variation in maize Trp abundance, root growth, and drought tolerance. Our results reveal that natural variation in the *ZmASB1* promoter, particularly InDel‐178, affects ZmDOF17 binding and promoter activity, thereby contributing to variation in *ZmASB1* expression, root Trp and IAA abundance, root growth, and drought responses. The enrichment of favorable *ZmASB1* alleles during maize breeding underscores their importance for adaptations to environmental conditions. These findings deepen our understanding of the interplay among amino acid metabolism, hormone signaling, and root development, while providing genetic insights and a candidate regulatory locus for further evaluation in maize root improvement and drought‐resilience breeding.

## 4 Methods

### 4.1 Plant Materials and Growth Conditions

This study included 192 maize inbred lines (Table S1) representing diverse germplasm and heterotic groups, including Tang SPT, Lvdahonggu, Lancaster, Reid, Pgroup, Tropic, and a mixed group. Seeds were visually inspected, and damaged, incompletely filled, shriveled, or unusually small or large seeds were discarded. The selected seeds were surface‐sterilized with 10% H_2_O_2_ for 30 min, rinsed thoroughly with distilled water, and soaked in saturated CaSO_4_ solution for 6 h. Seeds were then incubated on moistened filter paper in darkness at 28°C and 80% relative humidity for 2 days. Germinated seeds were transferred to two layers of germination paper (Anchor Paper Company, St. Paul, MN, USA), with eight seeds per roll. Seedling rolls were placed vertically in black cultivation boxes (39.5 × 29.5 × 22.5 cm) containing 7.5 L of nutrient solution, and the boxes were maintained in a greenhouse at Yangzhou University (32°30’N, 119°25’E). The nutrient solution was renewed every 2 days. After 2 weeks of growth, roots from three seedlings of each genotype were pooled to form one biological replicate. Two independent biological replicates were collected for amino acid extraction and liquid chromatography‐mass spectrometry (LC‐MS) analysis. Samples were immediately frozen in liquid nitrogen and stored at −80°C until analysis.

### 4.2 Amino Acid Determination and Analysis

The metabolomics data were obtained from our previously generated maize root metabolome dataset comprising 407 metabolites, including the 18 FAAs examined here [36]. The original sample preparation and LC‐MS procedures are summarized below. Approximately 200 mg of fresh root tissue was transferred to a 2 mL EP tube, and 600 µL methanol solution containing 2‐chlorophenylalanine (4 ppm) pre‐cooled to −20°C was added. The mixture was vortexed for 30 s, homogenized at 50 Hz for 60 s using a tissue grinder, and sonicated at room temperature for 15 min. After a 10‐min centrifugation (12,000 rpm and 25°C), the supernatant (300 µL) was collected and filtered through a membrane with 0.22 µm pores. The filtrate was transferred to autosampler vials for LC‐MS analysis. Each biological sample was analyzed by a single LC‐MS injection. A pooled quality‐control (QC) sample was prepared by combining equal 20‐µL aliquots from each study sample included in the analytical sequence. The pooled QC samples were injected after every 10 study‐sample injections to monitor instrument performance. Chromatographic separation was performed using a Thermo Vanquish system (Thermo Fisher, Shanghai, China) equipped with an ACQUITY UPLC^®^ HSS T3 column (150 × 2.1 mm, 1.8 µm; Waters). ESI‐MSn experiments were conducted using a Thermo Q Exactive mass spectrometer (Thermo Fisher), with spray voltages of 3.5 kV (positive) and −2.5 kV (negative). The full‐scan mode covered 81 to 1,000 *m*/*z* at 70,000 resolution, with sheath/auxiliary gas flow set at 30/10 arbitrary units and capillary temperature set at 325°C. Data‐dependent MS/MS used a collision energy of 30 eV. Raw MS data were first inspected using Analyst 1.6.3 software (SCIEX, USA) for spectral quality control. Raw data were converted to mzXML format using Proteowizard (v3.0.8789), and the XCMS R package (v3.1.3) was used for peak detection, filtration, and alignment. The resulting data matrix contained mass‐to‐charge ratio (m/z), retention time, and peak intensity information. Peak intensities were corrected for run‐order‐dependent analytical drift using pooled‐QC‐based support vector regression (SVR), and the corrected abundances were used for downstream analyses.

### 4.3 Statistical Analysis of 18 Amino Acids

Descriptive statistics for the 18 amino acids were calculated in R (v3.6.3). *H* ^2^ for each amino acid was estimated via a one‐way analysis of variance (ANOVA), with accessions as a random effect and two biological replicates providing the residual variation. *H* ^2^ was then calculated as follows: *H* ^2^ = *V*_g_/(*V*_g_ + *V*_e_), where *V*_g_ and *V*_e_ represent genetic and environmental variances, respectively. Amino acid data were z‐score normalized and visualized by hierarchical clustering using ComplexHeatmap and ggplot2 R packages. Pearson correlations among amino acids were visualized with the corrplot R package.

### 4.4 Genome‐Wide Association Study

The 192 maize inbred lines were re‐sequenced, which captured 13,361,037 SNPs with MAF ≥ 0.05. Marker pruning with PLINK used the parameters: ‐‐indep‐pairwise 10000 1 0.6, resulting in 499,885 effective markers. GWAS was performed with EMMA, including the first three principal components and a kinship matrix. The significance threshold was set at 2.00 × 10^−6^, corresponding to 1/499,885. Manhattan plots were generated using the R package CMplot. Significant SNPs for each amino acid were grouped into fQTLs using the PLINK parameters: ‐‐clump‐kb 50 and ‐‐clump‐r2 0.2, based on the estimated LD decay (Figure S5A). Clusters containing at least two significant SNPs were retained as putative fQTLs and represented by their lead SNPs. Genes located within 50 kb upstream or downstream of each lead SNP were annotated as fQTL‐associated genes.

### 4.5 FAA‐Gene Expression Correlation Analysis

For the 192 association panel lines, root transcriptome data were obtained from our previously published dataset. The transcriptome data was reanalyzed for FAA‐gene expression correlations and eGWAS [36, 37]. RNA extraction, library preparation, and sequencing procedures are summarized below. Total RNA was extracted using TRIzol reagent (Invitrogen) following the manufacturer's protocol, with RNA quality assessed using the RNA Nano 6000 Assay Kit and an Agilent Bioanalyzer 2100 system (Agilent Technologies, CA, USA). For each sample, 1.5 µg RNA was processed using a TruSeq paired‐end mRNA‐Seq Kit (Illumina) and sequenced on an Illumina HiSeq 2500 platform to generate paired‐end reads. Raw reads were processed (adapter trimming, poly‐N sequence removal, and quality filtering) to obtain clean reads, which were then aligned to the maize inbred line B73 reference genome RefGen_v3 using HISAT2 (v2.1.1). Uniquely mapped reads were retained for downstream analyses. Gene expression was quantified using featureCounts (v2.0.1) and normalized as fragments per kilobase of transcript per million mapped reads (FPKM) values.

For the correlation analysis, the expression levels of 37,219 genes obtained from RNA‐seq data were normalized using log_2_(FPKM + 1). Pearson correlations between the abundance of each FAA and gene expression were calculated using the corr.test function in the psych R package. Gene‐amino acid associations were considered significant when |*r*| > 0.2 and *p* < 0.01. Candidate genes were prioritized by integrating the correlation results with fQTL intervals. A gene was retained as a prioritized candidate only when it satisfied both of the following criteria: (i) it was located within the fQTL interval associated with a specific amino acid, and (ii) its expression level was significantly correlated with the abundance of that same amino acid. This integration yielded 17 prioritized candidate genes, which are listed in Table S6 together with their functional annotations.

### 4.6 Characterization of eQTLs for Candidate Genes

eGWAS was conducted for 17 prioritized candidate genes using the same SNP markers, EMMA model, principal components, kinship matrix, and significance threshold used for fQTL analysis. SNPs located within 20 kb upstream or downstream of a gene body were classified as cis‐eQTLs, whereas those outside this interval were classified as trans‐eQTLs, as described in previous studies [25, 66].

### 4.7 Construction of an Amino Acid Regulatory Network

An integrated network was constructed using 18 FAAs, 17 candidate‐gene expression profiles, 118 fQTLs, and 681 eQTLs. FAA‐fQTL and gene‐eQTL links were defined according to significant mGWAS and eGWAS associations, respectively. FAA‐gene links were retained when |*r*| > 0.20 and *p* < 0.01. The network was assembled and visualized using Cytoscape v3.10.2. Correlation coefficients were used to represent the direction and strength of associations, whereas *p*‐values represented statistical significance.

### 4.8 Mutant and Transgenic Materials

The *zmasb1* mutant line UFMu‐00576 was obtained from the Maize Stock Center. Genotypes were determined by nested PCR using Mu‐753F/Mu‐88R to amplify the WT allele and TIR6/Mu‐88R to amplify the transposon‐containing allele. PCR products were sequenced to verify the insertion site. For *ZmASB1* overexpression, the *ZmASB1* coding sequence was amplified from B73 root cDNA and cloned into the pCAMBIA1300221‐GFP vector under the control of the 35S promoter. Immature embryos of the B104 background were transformed, and T_0_ plants were verified by PCR. Stable overexpression lines were obtained after three generations of self‐pollination.

Root phenotypes were assessed using a paper‐roll hydroponic system. Eight seeds were placed on each roll, and six rolls were maintained in each bucket under a 14‐h light/10‐h dark photoperiod at 28/22°C. Buckets contained 2.3 L of nutrient solution, which was renewed every 2 days. At the two‐leaf‐one‐heart stage, five seedlings per bucket were treated as one biological replicate. Three roots were pooled for gene‐expression and amino acid analyses, and three independent biological replicates were analyzed. Root traits were measured manually and with WinRHIZO Pro 2004b.

### 4.9 Quantitative Real‐Time PCR Analysis

Total RNA was extracted from fresh roots with VeZol Reagent (Vazyme, Nanjing, China). cDNA was synthesized using HiScript II Q RT SuperMix for qPCR (+ gDNA wiper) (Vazyme). qRT‐PCR was performed with ChamQ SYBR qPCR Master Mix (Vazyme) on a CFX96 Real‐Time System (Bio‐Rad, Munich, Germany). Relative expression levels were calculated using the 2^−ΔΔCT^ method, with maize 18S rRNA as the reference gene.

### 4.10 Assay of Maize Seedling Drought Tolerance

Surface‐sterilized seeds were germinated as described above and transplanted into transparent plastic containers (length × width × depth, 30 × 10 × 10 cm) containing a soil mixture (vermiculite and substrate, 1:4). Thirty seedlings were arranged in six rows per container. Three rows of WT seedlings (left) and three rows of transgenic seedlings (right) represented one biological replicate. Drought stress was initiated by withholding water at 7 days post‐sowing. Samples and phenotypic measurements were performed when drought symptoms appeared (approximately 14 days after planting). Soil moisture was monitored using an HM‐WSYP soil moisture meter. Watering was resumed when the meter reading reached 0%, indicating that soil moisture was below the instrument's detection limit (approximately 25 days). Survival was recorded 3 days after rewatering. Six independent biological replicates were analyzed per line.

### 4.11 Exogenous Trp and IAA Treatments

For the hydroponic rescue assays, *zmasb1* seedlings were treated with Trp at concentrations of 0, 0.1, 1, or 10 µM, or with IAA at concentrations of 0, 0.1, 0.5, or 2.5 nM. WT seedlings were grown under mock‐treatment conditions and used as the reference control. Nutrient solutions were renewed every 2 days, and primary root length was measured after 1 week.

For the foliar treatment assays, WT and *zmasb1* seedlings were grown in pots containing vermiculite and substrate at a ratio of 1:4, with six seedlings per pot. At the one‐leaf‐one‐heart stage, *zmasb1* seedlings were sprayed once daily for 7 consecutive days with 100 µM Trp or 10 µM IAA supplemented with 0.1% Tween‐20. Mock‐treated *zmasb1* seedlings received 0.1% Tween‐20 alone, whereas WT seedlings were maintained without Trp or IAA treatment and served as the reference control. Watering was then withheld, and drought‐related traits were measured as described above. Six independent biological replicates were analyzed for each treatment.

### 4.12 Measurement of Physiological Parameters

Net photosynthetic rate (*P*_n_), transpiration rate (*T*_r_), and *g*_s_ were measured on the second fully expanded leaf using a portable photosynthesis system (LI‐COR 6800). WUE was calculated as *P*_n_/*T*_r_. For RWC, leaf fresh weight (FW) was recorded immediately after excision. Leaves were then rehydrated in distilled water at room temperature for 24 h to constant turgid weight (TW), oven‐dried at 105°C for 30 min, and further dried at 65°C to constant dry weight (DW). RWC was calculated using the formula [(FW–DW) / (TW–DW)] × 100%.

Three leaves per genotype from *zmasb1*, OE lines, and their respective WT controls were collected at the V1 stage for stomatal measurements. Each leaf was soaked in EMS/KOH buffer for 3 h, blotted dry, and coated with transparent nail polish on the abaxial surface. After 5‐10 min, imprints were collected with transparent tape and mounted on glass slides. Stomatal density was counted under a 10× objective in five fields per leaf, yielding 15 fields per genotype. Stomatal aperture was measured as the widest pore width using ImageJ under a 40× objective, with at least 35 fields per leaf and 200 stomata per genotype.

### 4.13 Determination of Trp and IAA Contents

Root tissue (150 mg) was extracted with 500 µL of extraction solution containing an internal standard, ground, and sonicated in an ice‐water bath. After incubation at −20°C and centrifugation, the supernatant was dried under nitrogen, reconstituted, and centrifuged again before LC‐MS/MS analysis on an AB Sciex Qtrap 6500+ system. Separation was performed on an ACQUITY UPLC^®^ HSS T3 column using a gradient of 0.1% formic acid in water and acetonitrile at 0.3 mL/min. MS detection was conducted in positive ESI mode with multiple reaction monitoring. Quantification used internal standard calibration curves (linear ranges: Trp 0.48‐5000 ng/mL, IAA 0.06‐5000 ng/mL; *r* ≥ 0.99). Final contents were calculated as *C*_c_ = (*C* × *V*_s_) / *m* × 1000, where *C*_c_ is the final concentration (pg/mg), *C* is the concentration from the standard curve (ng/mL), *V*_s_ is the reconstitution volume (mL), and m is the sample weight (mg). Each biological replicate consisted of roots pooled from three maize seedlings, with four biological replicates.

### 4.14 Virus‐Induced Gene Silencing Assay

Target fragments of *ZmASB1* or *ZmDOF17* were designed with the SGN‐VIGS website and cloned into pCMVZ‐2bN81, using the empty vector as a negative control. Constructs were transformed into *Agrobacterium tumefaciens* GV3101 and co‐cultured with pCMV‐F1 and pCMV‐FZ3. Cells were resuspended in infiltration buffer to OD_600_ = 1.0, and injected into *Nicotiana benthamiana* leaves. After 2‐4 days, infected leaves were ground in PBS, and the crude sap was injected into maize seeds on both sides of the embryo. Inoculated seeds were grown at 28/25°C under 16 h light/8 h dark. Silencing efficiency was assessed by qRT‐PCR at 12‐14 days post‐inoculation, and silenced plants were used for phenotypic and physiological analyses.

### 4.15 Transcriptome Analysis of *zmasb1* Mutant and OE Lines

Root samples from the *zmasb1* mutant, *ZmASB1* overexpression lines, and corresponding WT controls were collected under control and drought conditions. Four independent biological replicates were analyzed for each genotype and treatment. RNA‐seq library preparation, sequencing, read processing, genome alignment, and FPKM calculation followed the protocol described above. Differential expression analysis was performed using DESeq2 (version 1.52.0) implemented in the OmicShare platform (Genedenovo, Guangzhou, China) based on a negative binomial model. The Benjamini‐Hochberg method was applied to adjust *p*‐values for false discovery rate control. Genes with adjusted *p* < 0.05 and |log_2_ (fold change)| > 1 were considered differentially expressed. KEGG enrichment analyses were performed using KOBAS 3.0.

### 4.16 *ZmASB1*‐Based Association Analysis

We re‐sequenced an approximately 7,543‐bp genomic region encompassing *ZmASB1* (including approximately 2‐kb upstream and 0.5‐kb downstream sequences) in 169 of the tested inbred lines (Table S1). After filtering for MAF ≥ 0.05, we identified 284 SNPs and 101 InDels. Association analysis was performed using a mixed linear model (MLM) in TASSEL 5.0, using Trp abundance and *ZmASB1* expression as target traits. A threshold for significance of 1.30 × 10^−4^ (*p* = 0.05/*n*, *n* = 385) was applied. LD analysis was performed with LDBlockShow to visualize the LD block structure.

### 4.16 Detection of *ZmASB1* Promoter Activity

A 758‐bp promoter fragment upstream of the *ZmASB1* ATG that contained the significant variants, and three pairs of matched promoter fragments were generated: one containing the upstream variants, one differing only at InDel‐178, and one containing the downstream variant, were amplified from the two haplotypes and cloned upstream of N‐LUC in pGreen0800‐LUC. These fragments were analyzed using the same dual‐luciferase assay as described below. Constructs were introduced into *Agrobacterium tumefaciens* GV3101 (pSoup19) and transiently expressed in one‐month‐old *N. benthamiana* leaves. Empty pGreen0800‐LUC served as the control. After 3 days, infiltrated leaf discs (1‐2 cm) were collected, homogenized, and lysed. After centrifugation, the supernatant was collected and sequentially supplemented with firefly luciferase assay reagent and Renilla luciferase assay reagent. Luminescence signals were immediately measured using a Flx800 microplate luminometer. Relative activity (LUC/REN) was calculated using *Renilla* luciferase as an internal reference. Four biological replicates per sample were measured, each with three technical replicates.

### 4.17 Prediction and Verification of Functional Sites

PlantPAN3.0 was used to predict functional sites in the *ZmASB1* promoter and identify allelic variations affecting conserved transcription factor‐binding sites. To generate promoter fragments containing only the InDel‐178 site, 119‐bp (Hap1) and 114‐bp (Hap4) sequences flanking InDel‐178 were amplified and cloned into the *Spe*I site upstream of N‐LUC in pGreen0800‐LUC. The *ZmDOF17* CDS was cloned into the pGreen62‐SK vector using *BamH*I and *Sal*I. The constructs were co‐transformed into *A. tumefaciens* GV3101 (pSoup19) and co‐infiltrated into one‐month‐old *N. benthamiana* leaves using the same transient expression and luciferase assay procedures described above. Luminescence was measured 72 h after infiltration. Four biological replicates were analyzed per sample, with each biological replicate containing three technical replicates.

### 4.18 Electrophoretic Mobility Shift Assay

Protein expression strains were cultured in LB medium, and IPTG was used to induce expression. After lysis, the target protein was purified with an affinity column. Following SDS‐PAGE to assess expression and purity, the target protein was used for EMSA experiments. Synthesized probes (pro*ZmASB1* ^InDel+^ and pro*ZmASB1* ^InDel−^) (Table S9) were annealed using a PCR machine. Reagents were added sequentially, followed by a 20‐min incubation at room temperature. A 6% native‐PAGE gel was used, with pre‐electrophoresis performed to remove monomers, dimers, and polymerization initiators from the gel. Binding probes were mixed with EMSA loading buffer for electrophoresis and subsequent membrane transfer. After blocking with BSA, the membrane was incubated with blocking solution containing streptavidin‐HRP. The membrane was washed with PBST solution prior to imaging via ECL exposure.

### 4.19 Statistical Analysis

Unless otherwise stated, data are presented as mean ± standard deviation (SD). The exact sample size (*n*) and experimental units are reported for each analysis and, where applicable, for each figure panel. Detailed preprocessing procedures for the metabolomic and transcriptomic datasets are described in the corresponding Methods subsection. Comparisons between two groups were performed using two‐sided Student's *t*‐tests. Comparisons among more than two groups were analyzed using ANOVA, followed by Fisher's least significant difference (LSD) test. The significance level was set at *α* = 0.05. Statistical analyses were performed using GraphPad Prism 8.0, R, and the software packages specified in the relevant Methods sections. Exact *p*‐values or significance symbols are reported in the corresponding figures and figure legends.

## Author Contributions

Conceptualization was carried out by P.L., C.X., and Z.Y. Investigation was conducted by X.Y., H.W., X.Z., Y.W., S.F., W.R., T.Z., W.L., Y.Z., D.S., B.W., Y.T., H.G., Y.L., and Z.J. Writing (review and editing) was performed by P.L., C.X., Z.Y., X.Y., H.W., X.Z., Y.W., T.Z., S.F., W.L., W.R., Y.Z., D.S., B.W., Y.T., H.G., Y.L., and Z.J., while writing (original draft) was undertaken by X.Y., P.L., and C.X. Methodology was developed by P.L., C.X., and Z.Y. Resources were provided by C.X., Y.W., X.Z., T.Z., W.R., H.W., S.F., W.L., and X.Y. Funding acquisition was handled by P.L., C.X., and Y.W. Data curation was performed by P.L., H.W., X.Z., C.X., Z.Y., and X.Y. Validation was carried out by P.L., C.X., Z.Y., H.W., X.Z., and X.Y. Project administration was undertaken by P.L., C.X., and Z.Y. Formal analysis was conducted by P.L., H.W., X.Z., and X.Y. Software was developed by C.X., H.W., and X.Y. Visualization was performed by P.L., C.X., and X.Y.

## Funding

This work was supported by the National Key Technology Research and Development Program of the Ministry of Science and Technology of China (2022YFD1201804), the National Natural Science Foundation of China (32302654, 32572340, 32672680), the Open Research Fund Program of National Observation and Research Station of Agriculture Green Development (Quzhou, Hebei) (QZNS2025OF07), the Open Research Fund Program of State Key Laboratory of Nutrient Use and Management (KF2024‐9), the Innovative Research Team of Universities in Jiangsu Province, and the Priority Academic Program Development of Jiangsu Higher Education Institutions (PAPD).

## Conflicts of Interest

The authors declare no conflicts of interest.

## Supporting information




**Supporting File 1**: advs78656‐sup‐0001‐Figures.docx.

**Supporting File 2**: advs78656‐sup‐0002‐Tables.xlsx.

## Data Availability

Data and materials availability: All data needed to evaluate the conclusions in this paper are present in the paper and/or the Supplementary Materials. The raw reads of RNA‐seq from this study were deposited in the NCBI Sequence Read Archive (https://www.ncbi.nlm.nih.gov/sra) with the accession number PRJNA1551715.
