# Supplementary material for: Natural Variation in ZmASB1 Links Tryptophan Metabolism to Root Growth and Drought Tolerance in Maize
Source: Adv Sci (Weinh). 2026 Nov 10:e23671. Online ahead of print. doi: 10.1002/advs.202523671 (PMC13752245; doi:10.1002/advs.202523671)
Supplement: Supplementary file 1 [file ADVS-9999-e23671-s002.docx]

**Natural Variation in *ZmASB1* Links Tryptophan Metabolism to Root Growth and Drought Tolerance in Maize**

*Xiaoyi Yang^#1,2^*, *Houmiao Wang^#1,2^*, *Xiaomin Zhang^#3^*, *Yunyun Wang^1,2^*, *Yimei Tian^1,2^*, *Baoqing Wang^1,2^*, *Tianze Zhu^1,2^*, *Shuai Fang^1,2^*, *Wei Li^1,2^*, *Wenye Rui^1,2^*, *Yining Zhang^1,2^*, *Dan Sun^1,2^*, *Haoxin Gao^1,2^*, *Yuxuan Li^1,2^*, *Zhongtao Jia^4,5^*, *Zefeng Yang*^1,2^*, *Chenwu Xu*^1,2^*, *Pengcheng Li*^1,2^*

^1^Jiangsu Key Laboratory of Crop Genomics and Molecular Breeding/Key Laboratory of Plant Functional Genomics of the Ministry of Education, Agriculture College of Yangzhou University, Yangzhou 225009, China

^2^Jiangsu Co-Innovation Center for Modern Production Technology of Grain Crops/Jiangsu Key Laboratory of Crop Genetics and Physiology, Yangzhou University, Yangzhou 225009, China

^3^State Key Laboratory of Crop Stress Adaptation and Improvement, School of Life Sciences, Henan University, Kaifeng 475004, China

^4^National Observation and Research Station of Agriculture Green Development, Quzhou 057250, China

^5^State Key Laboratory of Nutrient Use and Management, College of Resources and Environmental Sciences, China Agricultural University, Beijing 100193, China.

\# These authors contributed equally to this work

* Correspondence: Pengcheng Li (pcli@yzu.edu.cn); Chenwu Xu (cwxu@yzu.edu.cn); Zefeng Yang (zfyang@yzu.edu.cn)

**Figure S1. Distribution of 18 free amino acids (FAAs) in maize roots.** Histograms showing the frequency distribution of the 18 amino acids detected in roots of 192 maize inbred lines. The x‑axis represents the abundance of each amino acid, and the y‑axis represents density. The dashed curve represents the theoretical normal distribution, and the solid green curve indicates the actual density distribution of the measured data.

**Figure S2. Correlations among free amino acids (FAAs) and their associations with root traits in maize. A.** Pairwise correlations among the 18 FAAs. Pearson correlation coefficients were calculated using genotype-level means (*n* = 192). Statistical significance was assessed using two-sided Pearson correlation tests. Correlation coefficients are displayed in the upper triangle, and circles summarize the correlations in the lower triangle. Green and brown indicate positive and negative correlations, respectively, with darker colors indicating larger absolute correlation coefficients. Asterisks indicate statistical significance. **B.** Correlation heatmap between 18 FAAs and 24 root traits in maize. Color intensity reflects the strength and direction of the correlation: green indicates positive correlations, while brown indicates negative correlations, with darker shades representing stronger correlations. Correlation coefficients (*r*) are displayed in each cell only for statistically significant correlations (*P* < 0.05); non-significant correlations are left blank. Correlation coefficients were calculated using genotype-level means, and significance was determined using two-sided Pearson correlation tests.

**Figure S3. Quantile-Quantile (Q-Q) plots for GWAS of 18 FAAs in maize roots**. Each panel displays the observed versus expected -log_10_(*P*) from the GWAS analysis for each of the 18 FAAs. The diagonal line represents the expected distribution under the null hypothesis.

**Figure S4.** **Manhattan plots of GWAS for 18 FAAs abundance in maize root**. Each panel displays the GWAS results for one of the 18 FAAs, with the x-axis representing chromosomal position across the ten maize chromosomes and the y-axis indicating -log_10_(*P*). The dashed horizontal line indicates the genome-wide significance threshold (*P* = 2.00 × 10^-6^, -log_10_(*P*) = 5.70), and SNPs exceeding the threshold are highlighted in red.

**Figure S5.** **Summary statistics of GWAS results for 18 FAAs in maize roots.** **A.** LD decay plot across the maize genome. The horizontal axis represents the physical distance (kb) between SNP pairs, and the vertical axis represents the squared correlation coefficient (*r*^2^). The LD decay distance in this population, estimated at *r*^2^ = 0.2, is approximately 50 kb. **B.** Number of eQTLs and candidate genes identified for each of the 18 FAAs based on eGWAS analysis and LD-based gene assignment. eQTLs were defined using a significance threshold of -log_10_(*P*) > 5.70, and candidate genes were assigned to eQTLs using the population-specific LD decay distance. **C.** Heatmap of Pearson correlations between root FAA abundance and gene expression across the 192 genotypes, calculated using genotype-level means. Columns represent amino acids, and rows represent genes. Green and brown indicate positive and negative correlations, respectively, with darker colors indicating larger absolute correlation coefficients. **D.** Number of genes significantly correlated with each of the 18 FAAs based on transcriptome correlation analysis. Statistical significance was determined using thresholds of |*r*| > 0.20 and *P* < 0.01.

**Figure S6.** **Manhattan plots of eGWAS for 17 amino acid-related candidate genes in maize root**. Each panel displays the genome-wide association results for the expression level of one candidate gene, with the x-axis representing chromosomal position across the ten maize chromosomes and the y-axis indicating -log_10_(*P*). The dashed horizontal line indicates the genome-wide significance threshold (*P* = 2.00 × 10^-6^, -log_10_(*P*) = 5.70), and SNPs exceeding the threshold are highlighted in red.

**Figure S7.** **Manhattan plots of GWAS for tryptophan abundance and *ZmASB1* expression level.** **A.** Manhattan plots of GWAS for tryptophan abundance on ten chromosomes of maize. **B.** Manhattan plots of GWAS for *ZmASB1* expression level on ten chromosomes of maize. For each plot, the x-axis represents chromosomal position, and the y‑axis represents -log_10_(*P*). The dashed horizontal line indicates the genome-wide significance threshold (*P* = 2.00 × 10^-6^, -log_10_(*P*) = 5.70), and SNPs exceeding the threshold are highlighted in red. S2_41652294 and S2_41652204 are co-located SNPs by Trp and *ZmASB1*.

**Figure S8.** **Schematic diagram of the Mutator transposon insertion site in the *zmasb1* mutant allele (UFMu-00576)**. The image is from the MaizeGDB genome browser for the *ZmASB1* locus. In the displayed gene model, gray rectangular boxes represent untranslated regions (UTRs), red rectangular boxes represent exons, and black lines represent introns. The positions of various UniformMu transposon insertions identified in the *ZmASB1* locus are shown along the gene model. The specific insertion corresponding to the *zmasb1* mutant allele, UFMu-00576, is highlighted with a red box.

**Figure S9. Quantification of root traits and shoot dry weight in maize seedlings. A-D.** Primary root length (PRL; **A**), seminal root length (SRL; **B**), lateral root length (LRL; **C**), and shoot dry weight (SDW; **D**) in wild-type (WT; W22) and *zmasb1* mutant seedlings (*n* = 30, 30, 15, and 3, respectively). **E-H**. PRL (**E**), SRL (**F**), LRL (**G**), and SDW (**H**) in WT (B104) and *ZmASB1* overexpression lines OE2 and OE7 (*n* = 30, 30, 15, and 3, respectively). Data are presented as means ± SD. Statistical significance was determined by two-sided Student’s *t*-tests (**A-D**) and analysis of variance (ANOVA), followed by LSD test for post hoc pairwise comparisons (**E-H**). *P* values are shown above the bars.

**Figure S10.** **Root and stomatal phenotypes of wild-type and *zmasb1* maize seedlings under drought stress.** **A**. Root dry weight (RDW) of WT and *zmasb1* seedlings after 3 d of rewatering following drought treatment (*n* = 6). **B-C**. Primary root length (PRL; **B**) and lateral root length (LRL; **C**) of WT and *zmasb1* seedlings under control (CK) and drought (Dr) conditions at 14 days after planting. Sample sizes were *n* = 8 for CK and *n* = 12 for Dr. **D**. Shoot fresh weight (SFW) of WT and *zmasb1* seedlings under CK and Dr conditions at 14 days after planting (*n* = 6). **E**. Stomatal density in 7-day-old WT and *zmasb1* seedlings. **F**. Quantification of stomatal aperture in 7-day-old WT and *zmasb1* seedlings (*n* = 200). Data are presented as means ± SD. Statistical significance was assessed using two-sided Student’s *t*-tests. *P* values are shown above the bars.

**Figure S11.** **Phenotypic and metabolic characterization of ZmASB1-silenced maize plants under drought stress.** **A**. Relative expression levels of *ZmASB1* in *ZmASB1*-VIGS (*n* = 49) and empty-vector (EV; *n* = 58) control plants. **B**. Survival rates of *ZmASB1*-VIGS and EV control plants after drought treatment and subsequent re-watering. Two independent VIGS experiments were performed. **C**. Representative images of root systems from *ZmASB1*-VIGS and EV plants grown under control (CK) and drought (Dr) conditions at 14 days after planting. Ten root systems per genotype and treatment were washed, pooled, and photographed. Scale bar, 5 cm. **D-E**. Total root length (TRL; **D**) and root dry weight (RDW; **E**) of *ZmASB1*-VIGS and EV plants under CK and Dr conditions at 14 days after planting. Data are presented as means ± SD (*n* = 16, 17, 16, and 16, respectively). **F-G**. Tryptophan (Trp; **F**) and indole-3-acetic acid (IAA; **G**) contents in roots of *ZmASB1*-VIGS and EV plants under CK and Dr conditions at 14 days after planting (*n* = 4). Data are presented as means ± SD. Statistical significance was assessed using two-sided Student’s *t*-tests. *P* values are shown above the bars.

**Figure S12. Root and stomatal phenotypes of wild-type and *ZmASB1* overexpression maize seedlings under drought stress.** **A**. Root dry weight (RDW) of WT, OE2, and OE7 seedlings after 3 d of rewatering following drought treatment (*n* = 6). **B-C**. Primary root length (PRL; **B**) and lateral root length (LRL; **C**) of WT, OE2, and OE7 seedlings under control (CK) and drought (Dr) conditions at 14 days after planting. Sample sizes were *n* = 9 for CK and *n* = 10 for Dr. **D**. Shoot fresh weight (SFW) of WT, OE2, and OE7 seedlings under CK and Dr conditions at 14 days after planting (*n* = 6). **E**. Stomatal density of 7-day-old WT, OE2, and OE7 seedlings. **F**. Quantification of stomatal aperture in 7-day-old WT, OE2, and OE7 seedlings (*n* = 200). Data are presented as means ± SD. Statistical significance was determined by ANOVA, followed by LSD test for post hoc pairwise comparisons. *P* values are shown above the bars.

**Figure S13.** **Differentially expressed genes and KEGG pathway enrichment in *ZmASB1* mutant and overexpression materials under control and drought conditions.** **A.** Number of up- and down-regulated DEGs in *zmasb1* compared to WT under CK and Dr conditions. **B.** Number of up- and down-regulated DEGs in OE2 and OE7 lines compared to WT under CK and Dr conditions. **C.** KEGG pathway enrichment analysis of four reciprocal-expression DEG intersection sets under CK and Dr conditions: (i) down-regulated in mutant and up-regulated in OE, (ii) up-regulated in mutant and down-regulated in OE, under both CK and Dr conditions. OE refers to the intersection of DEGs commonly identified in both OE2 and OE7 lines. The heatmap displays the Rich Factor, calculated as the ratio of DEGs annotated to a specific pathway to the total number of genes annotated to that pathway in the background genome. Color intensity represents the degree of enrichment, with darker green indicating higher enrichment. The numerical value within each cell represents the *P*‑value of the enrichment. Significance levels are indicated with * for *P* < 0.05, ** for *P* < 0.01, and *** for *P*<0.001. **D.** Transcriptomic changes in the Trp‑IAA biosynthesis pathway. The heatmap displays the expression levels of DEGs encoding enzymes involved in this pathway across different genotype and treatment combinations. Gene expression values represent the mean FPKM of four biological replicates, scaled to a range of 0-1 using Min‑Max normalization. For *zmasb1*, color intensity is shown in brown, with darker brown indicating higher expression levels. For OE samples, color intensity is shown in green, with darker green indicating higher expression levels.

**Figure S14. Effects of exogenous tryptophan (Trp) and indole-3-acetic acid (IAA) on the primary root growth defect of the *zmasb1* mutant under hydroponic conditions.** **A**. Primary root length (PRL) of WT and *zmasb1* seedlings, and of *zmasb1* seedlings supplemented with 0, 0.1, 1, or 10 μM Trp in the hydroponic medium. **B**. PRL of WT and *zmasb1* seedlings, and of *zmasb1* seedlings supplemented with 0, 0.1, 0.5, or 2.5 nM IAA in the hydroponic medium. Data are presented as means ± SD (*n* = 10 seedlings per genotype or treatment). Statistical significance was determined by ANOVA, followed by LSD test for post hoc pairwise comparisons. For rescue comparisons, each supplemented *zmasb1* group was compared with the untreated *zmasb1* group; the WT-versus-untreated-*zmasb1* comparison is also shown. *P* values are indicated above the bars.

**Figure S15.** **Effects of foliar application of exogenous tryptophan (Trp) and indole-3-acetic acid (IAA) on root growth and shoot biomass of maize seedlings under drought stress.** **A.** Root dry weight (RDW) of the four groups after 3 d of rewatering following drought treatment (*n* = 6). **B-C.** Primary root length (PRL; **B**) and lateral root length (LRL; **C**) under control (CK) and drought (Dr) conditions at 14 days after planting. Sample sizes were *n* = 8 for CK and *n* = 11 for Dr. **D.** Shoot fresh weight (SFW) under CK and Dr conditions at 14 days after planting (*n* = 6). Data are presented as means ± SD. Statistical significance was determined by ANOVA, followed by LSD test for post hoc pairwise comparisons. *P* values are shown above the bars.

**Figure S16. Phenotypic and metabolic characterization of *ZmDOF17*‑silenced plants under drought stress.** **A.** Relative expression of *ZmDOF17* and *ZmASB1* in *ZmDOF17*-VIGS (*n* = 109; CK and drought) and EV (*n* = 77; CK and drought) control plants. **B.** Correlation scatter plot between *ZmDOF17* and *ZmASB1* expression levels in roots of *ZmDOF17*‑VIGS and EV control plants. Each point represents an individual plant, with negative EV and positive samples included (*n* = 100, CK; *n* = 86, Dr). The brown and green line indicates the linear regression fit, with the shaded area representing the 95% confidence interval. **C.** Survival rates of *ZmDOF17*‑VIGS and EV plants after drought stress and re‑watering. Two independent VIGS experiments were performed. **D.** Representative images of root morphology of *ZmDOF17*‑VIGS and EV plants under CK and Dr conditions. Ten roots per treatment were washed, pooled, and photographed. Scale bar = 5 cm. **E-F.** Total root length (TRL; *n* = 18, 21, 10, and 10, respectively; **E**) and root dry weight (RDW; *n* = 18, 21, 24, and 16, respectively; **F**) of *ZmDOF17*‑VIGS and EV plants under CK and Dr conditions at 14 days after planting. Data are presented as mean ± SD. **G-H.** Trp (**G**) and IAA (**H**) contents in roots of *ZmDOF17*‑VIGS and EV plants under CK and Dr conditions. Data are presented as mean ± SD (*n* = 4). Statistical significance was determined by two‑sided Student’s *t*‑test.
